# Influence of a new botanical combination on quality of life in menopausal Spanish women: Results of a randomized, placebo-controlled pilot study

**DOI:** 10.1371/journal.pone.0255015

**Published:** 2021-07-21

**Authors:** Laura López-Ríos, Miguel A. Barber, Julia Wiebe, Rubén P. Machín, Tanausú Vega-Morales, Ricardo Chirino

**Affiliations:** 1 Department of Research, Development and Innovation, Nektium Pharma SL, Agüimes, Las Palmas, Spain; 2 Gynecological Division, Baren Clinic, Las Palmas de Gran Canaria, Las Palmas, Spain; 3 Department of Biochemistry, Molecular Biology, Physiology, Genetics and Immunology, Universidad de las Palmas de Gran Canaria, Las Palmas de Gran Canaria, Las Palmas, Spain; Fondazione Toscana Gabriele Monasterio, ITALY

## Abstract

**Background:**

This study was designed to evaluate the beneficial effects of a botanical extract combination containing soy isoflavone extract (100mg), *Aframomum melegueta* seed dry extract (50 mg), and *Punica granatum* skin dry extract (100mg) on health-related Quality of Life in healthy Spanish menopausal women with hot flashes, anxiety, and depressive symptoms using the validated Cervantes Scale.

**Methods and results:**

Fifty-seven outpatient women (45–65 years) with menstrual problems associated with climacteric syndrome were enrolled from April 2018 to April 2019 in the context of a prospective, placebo-controlled, double-blind study. Women were randomized to receive treatment with either the botanical combination (250 mg daily divided into two doses) or placebo for eight weeks. At the beginning and end of the study, health-related Quality of Life was assessed using the Cervantes Scale. Subjects treated with the botanical extract, compared to subjects in the placebo group, showed a significant improvement in the Global health-related Quality of Life score (38% [11.3–50.0]% vs. 18.8% [0–37.7]%; *P* = 0.04) on the Cervantes Scale and, specifically, in the menopause and health domain (13.6% [0–45.4]% vs. 40.7% [20.6–61.0]%; *P* = 0.05). By contrast, there were no significant changes in the psychic, sexuality, and couple relationship related domains of the Cervantes Scale. Patients who concluded the study did not report substantial side effects.

**Conclusion:**

Short-term intake of the botanical combination improved the Global Quality of Life of climateric women, according to the Cervantes Scale. Since this is a pilot trial, results should be analysed with caution.

**Trial registration:**

NCT04381026; ClinicalTrial.gov (retrospectively registered).

## Introduction

Menopause is a natural biological process related to the end of a woman´s reproductive life and is associated with physical symptoms such as vascular dysfunction (hot flashes, palpitations, and night sweats), psychological symptoms including mood changes, irritability, anxiety, or depression, and metabolic effects such as weight gain and slowed metabolism [1]. After the reproductive or premenopausal period, women usually experience menopausal transition between the ages of 45 and 55. Menopause is a process that lasts for 4–5 years, during which there are three main stages: perimenopause (alterations in the menstrual cycle and amenorrhea), menopause proper (involving the final menstrual period with at least 12 months with no menses), and post menopause (permanent cessation of period) [2,3]. In the first stage, the so-called perimenopause, estrogen levels are reduced and a high percentage of women experience a variety of symptoms—such as hot flashes, night sweats, or palpitations, as well as insomnia, anxiety, or depression—that affect their Quality of Life. The prevalence of such symptoms ranges from 74% in of women in Europe, 36–50% in North America, 45–69% in Latin America, and 22–63% in Asia [4].

During this early stage, the physical symptoms can lead to psychological impairment and reduce women’s perception of their health-related Quality of Life (HRQL); this is detectable soon after the onset of menopause [5,6]. Pre- and perimenopausal women with vasomotor symptoms are more prone to present psychological and somatic symptoms and stress, independent of life events, family dysfunction, or poor social support compared to postmenopausal women [7]. Specific psychometrics tools have been developed to evaluate the Quality of Life within different domains covering psychosocial, physical, sexual, and vasomotor aspects. These instruments are effective for identifying treatments that are best suited to these women and their particular situations. Several valid and reliable quality of life questionnaires such as the Menopause Specific Quality of Life Questionnaire (MENQOL) [8], the Menopause Rating Scale (MRS) [9], and the Kupperman Index [10] have been developed for the assessment of menopausal women´s symptoms. The Cervantes Scale (CS) is a HRQoL developed and validated for menopause in Spanish women aged between 45 and 64 years [11]. It contains 31 items distributed into four domains: menopause and health (15 items), psychology (9 items), sexuality (4 items), and couple relationship (3 items). The menopause and health domain include three subdomains: vasomotor symptoms, health, and aging and has been easily adapted for use as a complementary tool to other cultures [12]. The global CS score can range from 0 to 155 (best to worst perception) and a low score reflects a good perception of Quality of Life.

Although menopause is a transitory process, its physical and psychological symptoms greatly affect a woman’s work and family life. Therefore, many women seek some form of treatment. The most common option for reducing menopause symptoms is hormone replacement therapy (HRT); however, its use has been associated with an increased risk of developing breast cancer [13] and cardiovascular disease [14]. Other treatment options include low-dose anti-depressants such as Selective Serotonin Re-Uptake Inhibitors (SSRI), which are used to reduce hot flashes and improve mood [15]. Even though herbal remedies have been used for centuries, in recent years, several herbal preparations and botanical extracts have gained great popularity among older women compared to HRT due to their claimed ability to relieve menopausal symptoms and the perception of safety associated with natural products. In fact, several commercial bioactive-enriched extracts and preparations of black cohosh, valerian, evening primrose, angelica, chasteberry and St. John’s Wort (among others) are taken individually by climateric women to reduce menopause complaints [16]. However, the most common and evidence-based natural alternatives are phytoestrogens-rich supplements derived from certain herbs including soy, red clover, hops, licorice, rhubarb and flaxseed [17]. These weakly estrogenic botanicals may mimic the effects of endogenous oestrogens lost during menopause, which may contribute significantly to the alleviation of vasomotor symptoms [18–20]. To complement this beneficial effect, the combination of phytoestrogens with other botanicals with a selected profile of biological activities could be considered as a more effective approach to mitigate other alterations associated with climateric symptomatology [21,22]. The rationale for combining different botanical extracts is to find complementary extracts that alleviate physical and emotional problems and thereby increase the overall Quality of Life (QOL) of women in menopause. Therefore, a number of plants were carefully studied and some of them evaluated *in vivo* to combine the most suitable ones, with the intention of broadly covering most of the symptoms related to menopause. From this selection process, three botanicals were chosen for our study: *Aframomum melegueta* seeds (grains of paradise), *Glycine max* bean (soybean) isoflavones, and *Punica granatum* (pomegranate) skin.

*Aframomum melegueta*, also known by the common name “alligator pepper” or “grain of paradise”, belongs to the Zingiberaceae family. The seeds are widely used throughout the world as a valuable and commonly used culinary spice and as an agent for wide-ranging ethnobotanical uses. In African folk medicine, *Aframomum melegueta* seeds are used to treat constipation, rheumatism, fever, gastrointestinal disorders, and hypertension [23,24]. Mixed with salt and rubbed to the interior of the mouth, they are also used as a treatment for sleeping sickness [25,26]. Additionally, the seeds are chewed as a tonic for impotence and fatigue and as a stimulant, as well as to enhance endurance [25]. The above ethnobotanical evidence and the recent study in which Ojo et al. (2018) reported an anti-depressant [27] effect *in vivo* made *Aframomum melegueta* seeds a promising candidate for a menopausal product. In fact, low mood and depression associated with the menopausal period comprise variations in the serotonergic system, including increased serotonin clearance, a serotonin turnover and serotonin transporter affinity, as well as an increase in the serotonin receptor in different areas of the brain (i.e., prefrontal cortex and hippocampus). All these changes appear to be the result of an oscillation in the ovarian hormone concentration characteristics of climaterium.

*Glycine max* beans are one of the main plant sources of isoflavones which act as non-steroidal phyto-compounds with an estrogen-like effect, or phytoestrogen, widely used in traditional and alternative medicine to treat menopausal symptoms and alleviate vasomotor episodes [28]. Soybeans contain several isoflavone isomers, such as genistein, daidzein, and glycitein in the form of aglycones and genistin, daizine, and glycitin, in the form of glycones, which are the predominant phytoestrogen. The bioavailability of these molecules is modulated by the intestinal flora responsible for converting the isoflavones into their active metabolites (e.g., daidzein into equol). Although the biochemical structure of isoflavones differs from that of steroids, the few similarities between the molecules enable the low-affinity binding of isoflavones to the estrogenic receptors, mimicking the effect of endogenous estrogen and leading to a reduction in hot flashes and night sweats [29,30]. Furthermore, the role of isoflavones was evaluated by the Spanish Menopause Society (through a panel of experts) using the most reliable scientific evidence; the society concluded that, despite the existence of contradictory results between studies, “data on doses and genistein content have indicated beneficial effects on lipid profile, like antioxidants, as well as a mild decrease in hot flashes” in menopausal women [31].

Pomegranate (*Punica granatum* L) has been used since immemorial times in the traditional medicine of several civilizations, especially in the Middle East. It is a basic commercial crop full of bioactive compounds with several medical applications [32]. The skin of *Punica granatum* is very rich in punicosides, especially punicalagins, a polyphenolic compound with anti-inflammatory and potent anti-oxidant bioactivity that reduces oxidative stress, inhibits lipid peroxidation, and supports and improves the intestinal microbiota [33–35]. In addition, previous *in vivo* studies have shown the estrogenic, uterotrophic, and osteoprotective beneficial effects of *Punica granatum* extracts on ovariectomized rat models [36,37]. The profile of beneficial activities and the *in vivo* findings of protective estrogenic effects substantiate the use of pomegranate peel extract in the mitigation of menopause complaints.

As a placebo-controlled, double-blind, randomized study, this pilot clinical trial was designed to evaluate the beneficial effect of a rational combination of herbal extracts—made from *Glycine max* beans, *Aframomum melegueta* seeds, and *Punica granatum* skin—on the mitigation of perceived psychological, somatic, and socio-sexual symptoms in healthy Spanish menopausal women, adopting the validated Cervantes Quality of Life scale for the Spanish population.

## Materials and methods

### Electropharmacograms of Aframomum melegueta using spectral field power in conscious freely moving rats

To evaluate the effect of *Aframomum melegueta* extract on mood and depression, *in vivo* studies were performed by recording the field potentials of freely moving rats—a state-of-the-art technology that allows the analysis of brain waves via EEG [38]. Frequency analysis of signals from four brain regions allows the differentiation of clinically used botanicals and drugs with respect to their indication during the use of linear discriminant analysis [39]. This method can characterize CNS-active herbal preparations with respect to their potential clinical activity [40].

#### Animals

The study was performed at the preclinical laboratories of NeuroCode AG (Sportparkstr. 9, D-35578 Wetzlar/Germany) on nine 13-months-old adult male Fischer rats, day-night converted, weighing about 350–400 g, and provided by Charles River Laboratories, D-97633, Sulzfeld. The animals were allowed to acclimatize for at least 4 weeks before the start of the study. There was automatic control of light cycle, temperature and humidity. Animals were day-night reversed (12h/12h). Daily monitoring indicated that temperature and humidity remained within the target ranges of 22°C ± 2°C and 44% ± 5%, respectively. Cages, bedding, and water bottles were changed at regular intervals, i.e. every 2–3 days. Standard Diet (Nohrlin H10, Altromin, D-32791 Lage, Germany) was available ad libidum. All cages included Nestlets (Ancare) for environmental enrichment (replaced once every week). The animals had access to domestic quality mains water ad libitum. Animal handling and experimental procedures were carried out in accordance with the European Communities Council Directive (2010/63/EU) and the German Animal Welfare Act “Tierschutzgesetz” (BGBI IS.1105) and the National Institutes of Health Guide for Care and Use of Laboratory Animals. This study was approved by the local Animals Care and Use Committee of “Regierungspräsidium” Giessen, Hesse, Germany (Study Code: V54 19c 20 15h 01 NeuroCode Nr. 118/2014 A3/2014).

#### Method

The methodology of recording field potentials from a freely moving rat has been described in earlier publications [41]. Briefly, eight Fischer rats were implanted with a set containing four bipolar concentric steel electrodes into the brain within a stereotactic surgical procedure during anaesthesia with intraperitoneal injection of cocktail containing ketamine (70 mg/kg; Ketalar®, Pfizer, NY, USA), xylazine (13 mg/kg; Xilamax^®^; Bimeda-MTC, ON, Canada) and acepromazine (2 mg/kg; VETONE^®^, Idaho, USA). To alleviate postoperative pain, the animals were treated with a multimodal analgesic regimen post-surgery consisting of carprofen (5 mg/kg; Rimadyl^®^, Pfizer, NY, USA) and buprenorphine (0.02 mg/kg; Buprenex^®^, Reckitt Benckiser, Slough, England) given s.c. every 8 hours for 2 days. After two weeks for recovery from surgery, the transmitter was plugged in for adaptation and wirelessly sent local field potentials to a computer for frequency analysis. As the present technique results from several years of refinement and experimentation, no rodent died perioperatively as a consequence of the intervention. For testing brain wave modulation, a dose of 50 or 100 mg/kg of AME was administered by gavage. A negative control consisting of 0.9% NaCl solution (1mg/kg) was included. A crossover design with at least 3 days of washout between administrations was used. Recording of field potentials was performed for 2 h after an initial pre-drug baseline of 45 minutes. Transmitted data were processed by Fast Fourier Transformation (FFT) and spectral power was documented for eight frequency ranges (delta, theta, alpha1, alpha2, beta1a, beta1b, beta2, and gamma) within frontal cortex, hippocampus, striatum, and midbrain reticular formation at 1-hour intervals. Thirty-two variables (4 electrode positions x 8 frequency ranges) were fed into a linear discriminant analysis. The results of the linear discriminant analysis were documented by depicting the results from the first three functions into space (x, y, and z coordinates) and by depicting the results from the second three functions into three colours (RGB mode as used in TV). CNS active pharmaceuticals and botanicals typically show a specific EEG “fingerprint” (the electropharmacogram) and the spectral signature of the test substance can be compared to those of a pre-evaluated drug. A test substance with a spectral signature similar to that of a pre-tested drug or botanical can be expected to have similar CNS activities. In this case, the results obtained from AME were compared to a reference compound—Rolipram, a well-known antidepressant. Statistic evaluation in comparison with the control was performed using a non-parametric Wilcoxon rank-sum test.

### Evaluation of health-related quality of life according to the Cervantes Scale—pilot clinical trial

**Fig 1:**
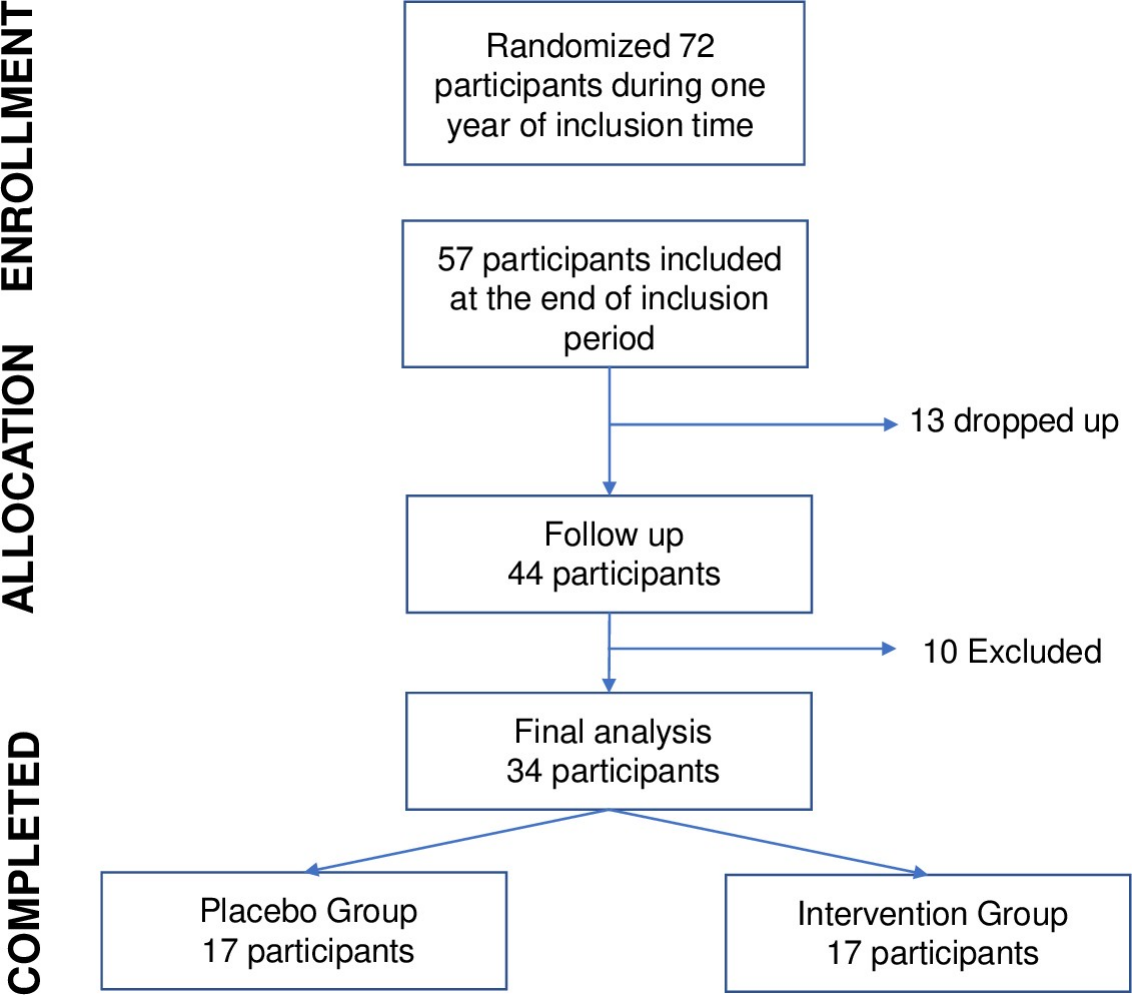
Trial enrollment profile. In total, 72 women with menopause were randomized during inclusion time period, 57 met the inclusion criteria and were enrolled in the study. Some participants resigned and others were excluded from the trial for various reasons (see flow diagram for details).

The study protocol was approved by the Ethics Committee of the University of Las Palmas de Gran Canaria (CEIH-2017-06) and was carried out according to the principles of the Helsinki Declaration. The trial was retrospectively registered at ClinicalTrial.gov (NCT04381026); the delay in registration was partly due to lack of knowledge of the need to pre-register pilot studies prior to subject recruitment. However, the authors confirm that all ongoing and related trials to this botanical combination are registered. All participants signed the written informed consent form which was stored in a secured locker accessible only to designated investigators. All the participants were Spanish women. Enrollment was conducted by the head physician of the Clinica Baren (Las Palmas de Gran Canaria, Spain) from April 2017 to April 2018, according to a pre-set randomization table of a two-arm design. The subjects were asked to freely participate in the study during a routine gynecological visit. Fifty-seven menopausal women who met the following inclusion criteria were enrolled (Fig 1): healthy women between 45–55 years, with not more than five years since the onset of menopause and with an initial Cervantes Scale (CS) value ≥53. The exclusion criteria were the use of Hormone Replacement Therapy (HRT), frequent intake of soy products, the taking of lipid-lowering drugs in the last year, a family history of endocrine cancer, the presence of hormonal pathologies, and surgery-induced menopause.

At the initial study visit, the subjects were randomized to receive treatment with the multi-herbal formula or the placebo at a dose of 250 mg daily, divided into two 125 mg pills taken at breakfast and dinner for eight weeks. The pills were similar in shape and size for both groups. Randomization was performed beforehand by a statistician (not involved in the study) in a 1:1 ratio without blocking using computer-generated random numbers. The randomization list was kept closely confidential. A person from the clinic was designated to randomize participants and package the botanical/placebo pills with labels. This person was not involved in any other stage of the study. Subjects were instructed to maintain their usual lifestyles. The 32-item CS questionnaire previously described and validated by Palacios et al (2004) was completed in the clinic by the participant prior to the start of the study protocol and no later than three days after the end of the treatment. The percentage of variation in the Cervantes Scale throughout the treatment was calculated as 100 X (Initial Value–Final Value)/Initial Value. To improve the validity of the data, women returned de bottles at the last visit and unused capsules were counted and recorded on the relevant case report form. Thirteen patients were excluded because they did not complete the entire study or did not respond to three or more questions (≥10%) of the Cervantes Scale. Women with a significant disparity between their baseline and final scores on the Cervantes Scale—specifically, a final score less than 30% of baseline (>70% improvement in the percentage of variation; n = 10)—were discarded because these changes were so drastic that could hardly be attributed to placebo or product treatment. Thus, the number of women included in this study was 34 (17 taking the placebo and 17 taking the extract). The participants, the head physician, the nurse and the statistician remained blinded from the identification of the two treatment groups until the completion of the study.

### Botanical extract preparation

*Aframomum melegueta* seed extract (AME) from Nektium Pharma SL (Spain) was obtained after 30% hydroethanolic extraction of finely powdered dried seed and concentration by solvent removal by rotatory evaporation before a final spray drying step. Standardized *Glycine max* bean dry extract (GME) containing 40% of total isoflavones was purchased from Layn Natural Ingredients, Inc (China). *Punica granatum* extract (PGE) was obtained from the skin by hydroalcoholic extraction, followed by purification and standardization to 40% total punicosides (Nektium Pharma SL (Spain)). The multi-herbal formula used in the randomized pilot clinical trial resulted from the combination of AME (50 mg), GME (100 mg), and PGE (100 mg). Pills containing 125 mg of the multiherbal formula or placebo (Maltodextrin; 125 mg) were properly manufactured and placed in hermetically sealed containers (40 pills per bottle; 3 bottles/participant; covering 8 weeks of treatment).

### Statistic and sample size

Statistical analyses were performed with SPSS for Windows, version 25 (IBM, Armonk, NY). Continuous variables were represented by the mean and standard deviation (SD) when data follow a normal distribution, or as the median and range when distribution deviated from normality. Means were compared by the Student’s t-test, and medians by the Wilcoxon rank-sum test for independent data. Statistical significance was set at p *<* 0.05. A priori power calculations estimated that a minimum of 36 participants in each group is sufficient to detect a 15% change in CS score with 80% of statistical power (alpha =  0.05). A dropout rate of 15% was included.

## Results

### Electropharmacograms of Aframomum melegueta using spectral field power in conscious freely moving rats

**Fig 2:**
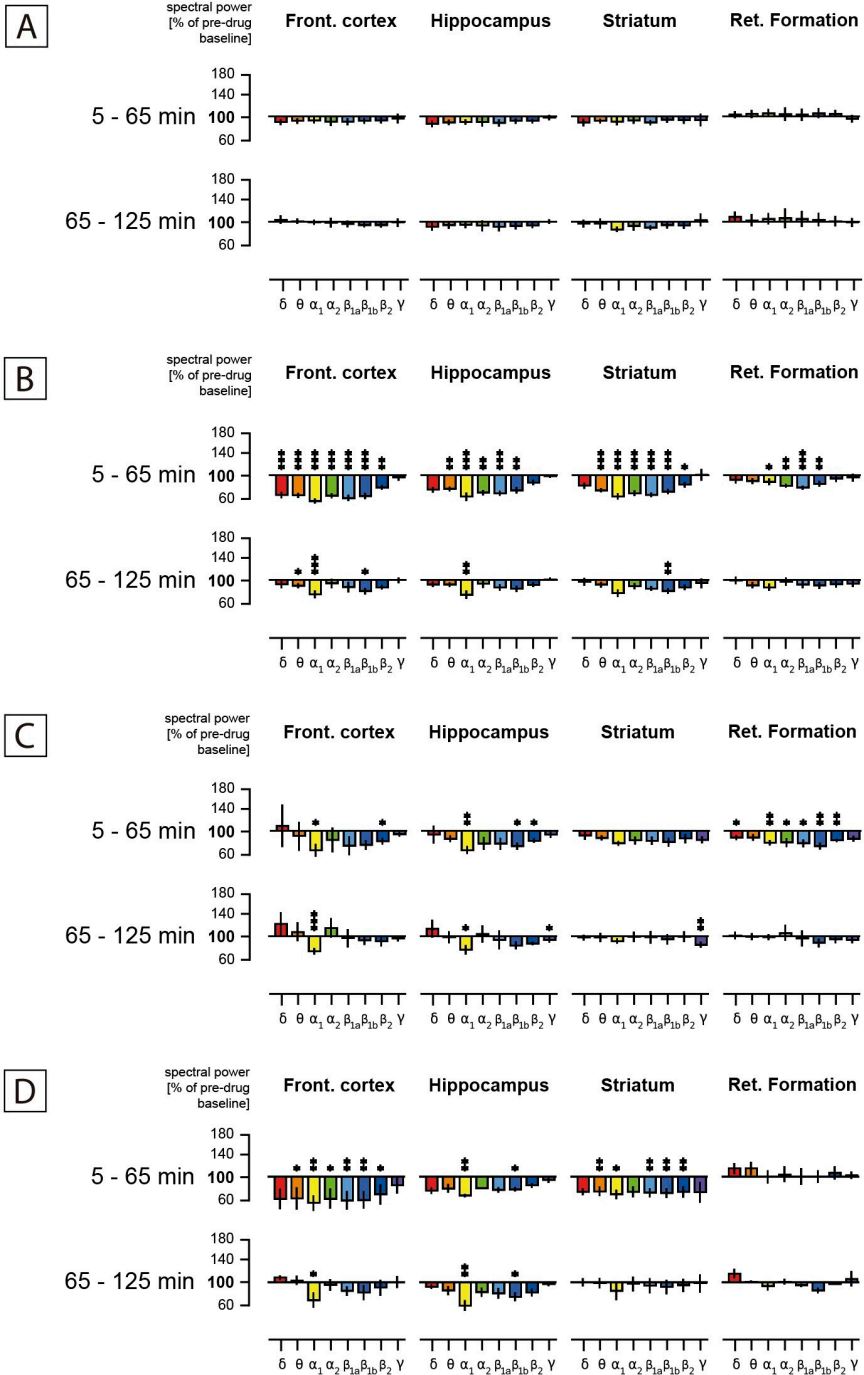
Effect of administration of vehicle (A), Rolipram 1 mg/kg (B), and *Aframomum melegueta* ethanolic extract (AME) at doses of 50 mg/kg (C) and 100 mg/kg (D): Timeline. Time dependence of spectral power changes (Ordinate) in % of 45 min lasting pre-drug baseline values in four brain regions of a freely moving rat in the presence of different tests. Frequency ranges are depicted as coloured bar graphs on the abscissa representing delta (red), theta (orange), alpha1 (yellow), alpha2 (green), beta1a (light blue), beta1b (blue), beta2 (dark blue), and gamma spectral power (violet) from left to right within the four brain areas as mentioned at the top of the graph. Statistical significance compared to control (vehicle) is documented with stars: * = p<0.10; ** = p<0.05; *** = p<0.01.

**Fig 3:**
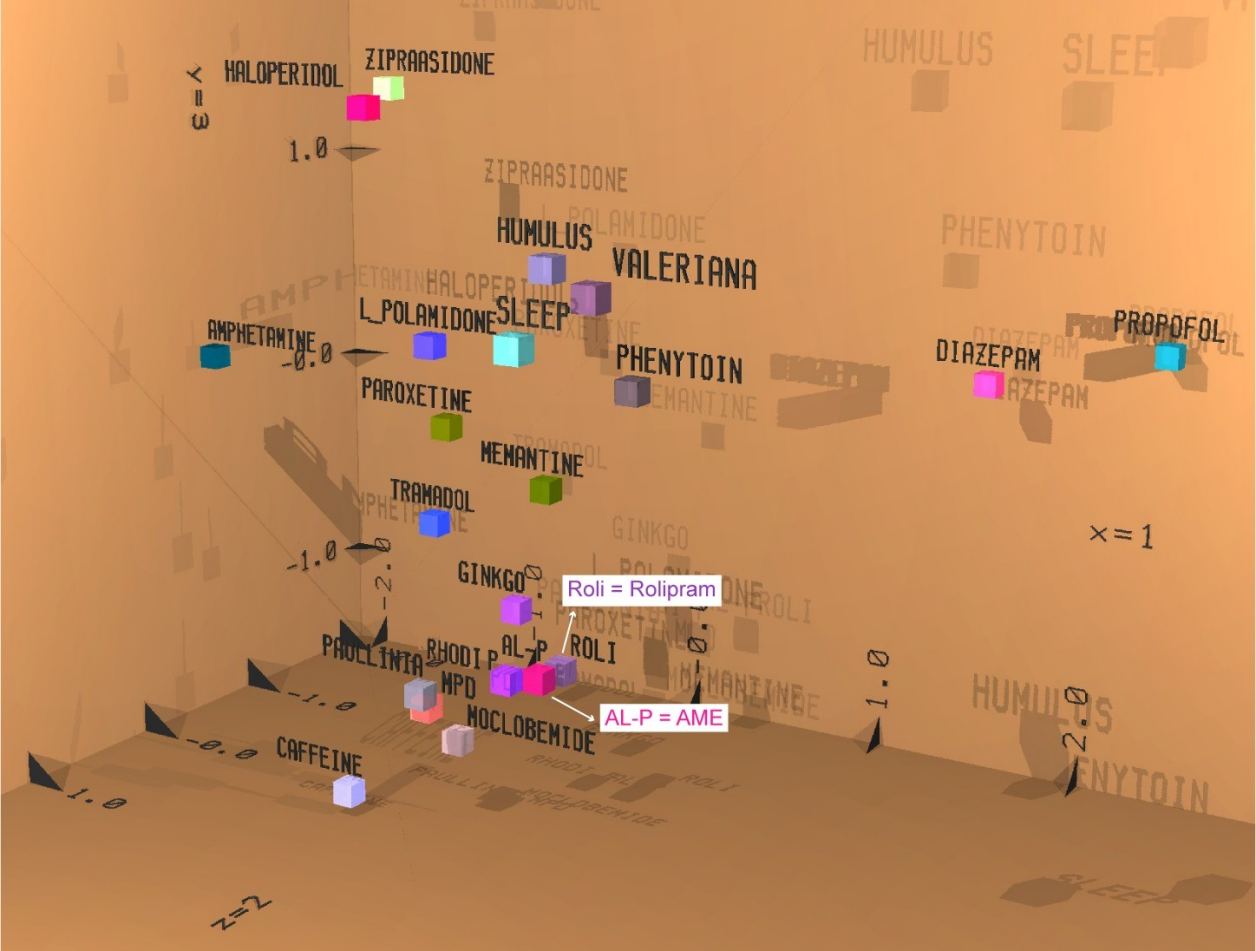
Discriminant analysis of electropharmacograms. Comparison of the electropharmacogram of the ethanolic extract of *Aframomum melegueta* seeds (AME; 100.0 mg/kg) administered orally. Synthetic drugs were given by intraperitoneal injection. The patterns of the reference drugs providing similar spectral frequency changes for similar clinical indications according to the results of the first three discriminant functions are positioned in close proximity (see x, y, and z coordinates). A high similarity with respect to space and colour signalizes similar net effects with respect to clinical indication. Data are from the first recording period after administration.

As expected, the oral administration of the vehicle (0.9% NaCl) resulted in only very minor changes in spectral power within the four brain areas (Fig 2A). By contrast, the oral gavage of 50mg/kg of an ethanolic extract of *Aframomum melegueta* resulted in a statistically significant attenuation of alpha1 spectral power, which lasted two hours after recording in the frontal cortex and hippocampus (Fig 2C). This result was confirmed with a higher dose of 100.0 mg/kg *Aframomum melegueta* extract, which also resulted in a significant attenuation of alpha1 spectral power in the cortex, hippocampus, and striatum during the first hour and in the cortex and hippocampus during the second hour. In addition, there was a statistically significant attenuation of alpha1, beta 1a, and beta1b spectral power during the first hour in the cortex and striatum (Fig 2D). Notably, the reference drug Rolipram (Fig 2B), a selective phosphodiesterase IV inhibitor with antidepressant and neuroprotective activity, produced a similar electropharmacogram profile [42]. Discriminant analysis also revealed a close position of the action of *Aframomum melegueta* extract (AL-P) to Rolipram (Fig 3), providing *in vivo* evidence of antidepressant-like activity. Interestingly, *Aframomum melegueta* extract was also in close proximity to the herbal root extract of Rhodiola rosea (Rhodi P; Fig 3)—a medicinal shrub with known “adaptogenic” properties, which has been reported to relieve stress and antidepressant activity [43,44].

### Evaluation of health-related quality of life according to the Cervantes Scale—pilot clinical trial

After the physical examination of all participants in this pilot clinical trial, the specialist physician confirmed the participants’ health and diagnosis of premenopause or menopause. Table 1 shows the characteristics of the patients enrolled in the two treatment groups and the Cervantes scores in both groups before the start of the clinical trial.

**Table 1:** Baseline characteristics of the women enrolled in the study.

Physical parameters	Placebo (n = 17)	Herbal extract (n = 17)
Age (years)	49.2 ± 4.5	49.2 ± 2.9
Weight (kg)	75.6 ± 11.8	72.6 ± 9.0
Body mass index (kg m^-1^)	28.3 ± 5.1	26.3 ± 3.6
Waist (cm)	90.8 ± 14.0	87.7 ± 11.5
Cervantes Scale score		
Global	58 [54–71]	58 ([55–74]
Menopause and health	41 [35–47]	37 [32–44]
Psychic domain	9 [6–13]	10 [8–15]
Sexuality	8 [6–13]	14 [9–16]
Couple relationship	3 [2–8]	4 [1–9]

Physical parameters are summarized as mean (±SD) and Cervantes Scale score as median with ranges.

**Fig 4:**
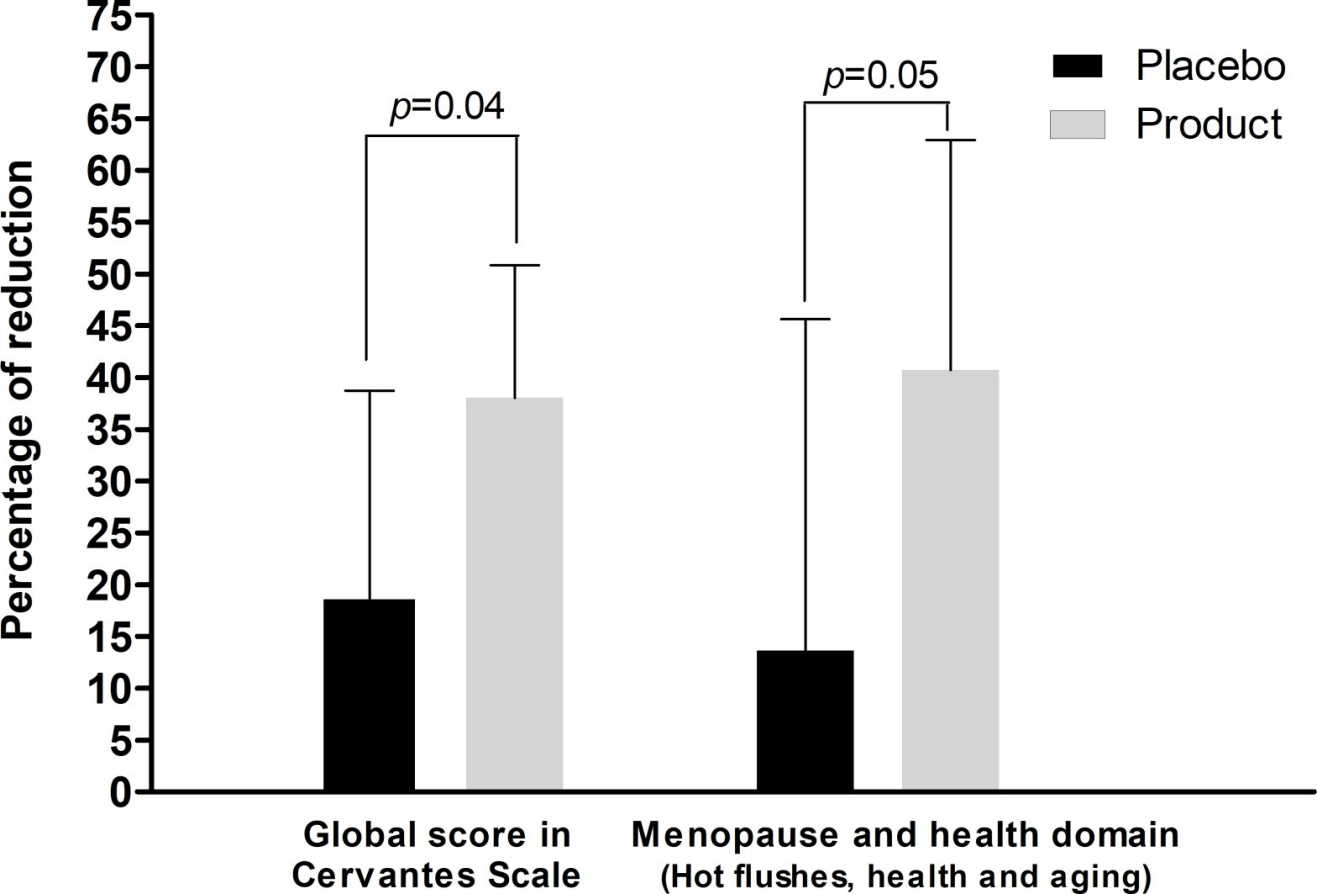
Self-reported Cervantes Scale. Percentage (%) of change in the overall Quality of Life score (global) and in the menopause and health domain (hot flashes, health, and aging) after treatment with the botanical extract combination (*Glycine max* seed isoflavones + *Aframomum melegueta* seed extract + *Punica granatum* skin extract) or placebo. Data are expressed as median ± range.

To evaluate the improvement of Quality of Life after the trial, we generated a variable to quantify the percentage reduction between after treatment and before treatment (*Variation% = 100 x (initial value-final value)/initial value*) for both the global score and for each domain. After 8 weeks of treatment, the percentage reduction in the global score was significantly higher in the botanical extract-treated group compared to the placebo group (38.0% [10.7–50.9]% vs. 18.8% [0–38.7]%; p = 0.041) and marginally significant for the menopause and health domain (40.7% [16.9–62.9]% vs. 13.6% [0.0–45.6]%; p = 0.053) (Table 2 and Fig 4), representing the domain of vasomotor symptoms, hot flashes, sleep quality, and aging perception.

**Table 2:** Percentage improvement in global score and domains between groups after treatment using the self-reported Cervantes Scale.

Cervantes scale domains	Placebo (n = 17)	Herbal extract (n = 17)	*P value*^a^
**Global (%)**	18.8 [0.0 to 38.7]	38.0 [10.7 to 50.9]	0.04
**Menopause and health (%)**	13.6 [0.0 to 45.6]	40.7 [16.9 to 62.9]	0.05
**Psychic domain (%)**	23.1 [0.0 to 75.6]	42.9 [14.3 to 79.4]	0.25
**Sexuality (%)**	0.0 [0.0 to 6.3]	0.0 [0.0 to 25.4]	0.27
**Couple relationship (%)**	0.0 [0.0 to 8.3]	0.0 [0.0 to 80.0]	0.11

Cervantes scale domains are summarized as median with ranges.

^a^P values for intergroup differences in Cervantes scale domains calculated by Wilcoxon rank-sum test.

Throughout the pilot study, no appreciable changes in anthropometric and serum biochemical parameters (glucose, lipids, hepatic enzymes, etc.) were observed and no participants experienced adverse side effects while taking the herbal extract or placebo. (Data available in https://osf.io/a359t/).

## Discussion

Two conclusions can be drawn from the results presented above. First, the AME included in the botanical combination is able to elicite substantial changes in the brain electrical activity, similar to those elicited by rolipram or Rhodiola rosea extract; and, second, the herbal combination is able to alleviate menopausal symptoms.

In our randomized, double-blind, placebo-controlled pilot study, the overall scores CS scores showed that the improvement in the perception of quality of life was greater in those treated with the botanical group than in the placebo group. This positive effect was specifically observed in the menopause and health domain, which evaluates vasomotor symptoms such as hot flashes, headaches, feeling tired due to a lack of sleep, and perception of aging through joint pain, dry skin, or urinary incontinence. The association was not observed in the psychic, sexuality, and couple relationship domains.

According to the results of the CS questionnaire, the botanical combination alleviated menopausal complaints encompassing the three main disorders: vasomotor symptoms, lack of sleep and energy, and aging perception. The product’s ability to reduce vasomotor symptoms may be predominantly associated with soy isoflavones and their ability to act as lower affinity lower, potency phytoestrogens, operating primarily as a selective modulator of the β-estrogen receptor [45]. In fact, despite the disparity among the authors regarding the effects of phytoestrogens on menopausal symptoms, several clinical trials have reported that soy isoflavones mitigate vasomotor symptoms [46], though with limited or no effects on cognition and other menopause-related symptoms [47]. Currently, isoflavones represent one of the most popular and safest alternative therapies to support menopausal complaints, although some contradictory results have been published in clinical studies [29,48]. Furthermore, the observed improvement in the CS health subdomain suggests a favourable effect on feelings of tiredness and sleepless, which could be tentatively associated with the *in vivo* antidepressant effect exerted by the ethanolic extract of *Aframomum melegueta* seeds using validated mice models [27]. This hypothesis is supported by the results obtained in an electropharmacogram using spectral field power in conscious freely moving rats, in which AME (Fig 2) can be classified as having an anti-depressive potential according to its spectral power profile, with a general attenuation of all frequencies dominated by a decrease in alpha 1 power, followed by delta and alpha 2 power. This EEG modulation profile indicating a positive effect on serotonergic neural transmission was very similar to the EEG of several antidepressant drugs in previous studies [39,49]. Serotonergic transmission in the limbic system and emotional functions are potentiated by estrogen, which positively modulates autonomic function, affections, mood, and the sleep-wake cycle [50]. Thus, the positive effect exerted by the herbal combination on the health subdomain may be mediated primarily by AME stimulation of the serotonergic system that supports serotonin levels. These preliminary *in vivo* results indicate that AME is a good candidate for completing the formulation that supports the menopause process, a complex condition in which symptoms of low mood, fatigue, and depression are very common among women.

Finally, the improvement in women’s perception of aging may be attributed to pomegranate skin extract, a botanical preparation rich in punicalagins, which are phenolic ellagitannins with notable antioxidant, anti-inflammatory, and immunomodulatory properties [51,52].

There was limitation in our study. The results of the evaluation interventions were conducted using only self-report data and could be susceptible to the placebo effect. In fact, it has been reported that menopausal women are prone to the placebo effect; for instance, some studies have previously reported that placebo groups self-report an improvement in vasomotor symptoms ranging from 20–60% [53]. In this clinical trial the possible prevalence of the placebo effect, together with interindividual variation in the bioavailability and pharmacokinetic parameters of nutraceuticals may be, at least partly, causal factors of the high variance in the Cervantes Scale domain data (Table 2), both in placebo- and herbal-treated groups [29]. In this context, the use of highly standardized botanicals preparations and the use of objective evaluation methods for interventions addressing menopausal symptoms would be desirable; for example, the use of specific skin conductance devices for the hot flash analysis as a physiological indicator of vasomotor symptoms.

## Conclusion

This pilot clinical trial provides preliminary evidence that eight weeks of treatment with the botanical extract combination could be an alternative for improving the overall Quality of Life in climacteric Spanish women according to the Cervantes Scale, particularly in the perception of hot flashes, health, and aging. Nevertheless, a larger prospective RCT study using objective assessment methods is required to provide sustainable evidence of the efficacy of the botanical preparation specifically in ameliorating climacteric symptoms. Since this is a pilot study, results should be analysed with prudence.

## Supporting information

S1 File Consort checklist. (DOC)

S2 File Study protocol Spanish Ethics Committee. (DOCX)

S3 File Annexes to protocol. (DOCX)

S4 File Study protocol Spanish Ethics Committee (in Spanish). (DOCX)

S5 File Annexes to protocol (in Spanish). (DOCX)
