# Supplementary material for: Influence of a new botanical combination on quality of life in menopausal Spanish women: Results of a randomized, placebo-controlled pilot study
Source: PLoS One. 2021 Jul 21;16(7):e0255015. doi: 10.1371/journal.pone.0255015 (PMC8294509; doi:10.1371/journal.pone.0255015)
Supplement: S2 File — (DOCX) [file pone.0255015.s002.docx]

Anexos

Estudio del efecto de un suplemento nutricional con isoflavonas sobre la sintomatología del climaterio en mujeres sanas

**Cod. Proyecto: PRO_WH_HCT_2016.01**

ÍNDICE

[I. ANEXO 1: Información al participante (7 hojas) 3](#_Toc473728091)

[II. ANEXO 2: Consentimiento informado (1 hojas) 10](#_Toc473728092)

[III.ANEXO 3: Cuestionario de salud (2 hojas) 11](#_Toc473728093)

[IV. ANEXO 4: Recomendaciones dietéticas (2 hojas) 13](#_Toc473728094)

[V. Anexo 5: Cuestionario de alimentación (consumo de soja) (1 hoja) 15](#_Toc473728095)

[VI. Anexo 6: Escala Cervantes (2 hojas) 16](#_Toc473728096)

[VII. ANEXO 7: Cuestionario de seguimiento/finalización (2 hojas) 18](#_Toc473728097)

[VIII. ANEXO 8: Cuestionario telefónico (1 hoja) 20](#_Toc473728098)

[IX. ANEXO 9: Diarios de seguimiento (2 hojas) 22](#_Toc473728099)

# ANEXO 1: Información al participante (7 hojas)

**TÍTULO DEL ESTUDIO**:

Estudio del efecto de un suplemento nutricional con isoflavonas sobre la sintomatología del climaterio en mujeres sanas

**CÓDIGO DEL PROMOTOR:** PRO_WH_HCT_2016.01

**PROMOTOR:** Nektium Pharma SL

**INVESTIGADOR PRINCIPAL**: Dr. Miguel Barber (Clínica Baren), Dr. Ricardo Chirino (ULPGC)

**CENTRO:** Clínica Baren

**Responsable en el centro:** Dr. Miguel Barber

**Responsable Nektium Pharma** **SL**: Dra Laura López Ríos

**Introducción**

Nos dirigimos a usted para informarle sobre un estudio de investigación en el que se le invita a participar. El producto que se va a ensayar es un suplemento nutricional, y el estudio ha sido aprobado por el Comité de Ética de Investigación Humana de la Universidad de Las Palmas de Gran Canaria (ULPGC). Nuestra intención es tan solo que usted reciba la información correcta y suficiente para que pueda evaluar y juzgar si quiere o no participar en este estudio. Para ello lea esta hoja informativa con atención y nosotros le aclararemos las dudas que le puedan surgir. Además, puede consultar con las personas que considere oportuno.

**Participación voluntaria**

Debe saber que su participación en este estudio es voluntaria y que puede decidir no participar o cambiar su decisión y retirar el consentimiento en cualquier momento, sin que por ello se altere la relación con su médico ni se produzca perjuicio alguno en su tratamiento.

**Descripción general del estudio**

Tanto los hombres como las mujeres experimentan un declive tanto físico como mental con el paso de los años. En el caso de la mujer, la pérdida de los estrógenos se asocia a un tipo de sintomatología concreta conocida como climaterio, que puede reducir su calidad de vida. El **Climaterio** se conoce como el *periodo de transición* que se prolonga durante años, tanto antes (perimenopausia), como durante (menopausia) y después de la [menopausia](https://es.wikipedia.org/wiki/Menopausia) (postmenopausia), como consecuencia del agotamiento [ovárico](https://es.wikipedia.org/wiki/Ovario), asociado a una disminución en su capacidad para producir [hormonas](https://es.wikipedia.org/wiki/Hormona), [folículos](https://es.wikipedia.org/wiki/Fol%C3%ADculo_ov%C3%A1rico) y [ovocitos](https://es.wikipedia.org/wiki/Ovocito). La franja de edad más habitual suele ser de los 45 a los 55 años, siendo los 51 años la edad media de la menopausia en nuestro país (Instituto Nacional de Estadística, 2011). Es un período de involución en la que se produce la pérdida paulatina de estrógenos y se acompaña de una serie de manifestaciones físicas y emocionales relacionadas con cambios biológicos y sociales (sofocos, ganancia de peso, insomnio, irritabilidad, descenso de la libido, etc.). Todas las manifestaciones no son comunes a todas las mujeres que están pasando por esta etapa, sino que dependen de cada mujer.

La **perimenopausia** se extiende desde el momento en que aparecen las primeras alteraciones del ciclo menstrual hasta el año siguiente al cese definitivo de la menstruación. Comienza con un aumento del sangrado vaginal (sangrado por más de 7 días), seguido de un espaciamiento entre las menstruaciones (amenorrea de más de 60 días) y por último 12 meses seguidos sin menstruación. La duración puede variar entre dos y cinco años. Se produce la supresión paulatina de la actividad ovárica, tanto en la cantidad como en la calidad de los ovocitos, por lo que es un periodo de baja fertilidad con alteración en los niveles de estrógenos que tienden a desaparecer. La **menopausia** es el periodo en la vida de la mujer en el que se carece de estrógenos por el cese de la actividad ovárica y, por tanto, ya no es fértil. Se considera que se inicia tras un año entero sin menstruación. Pasado ese año se habla de **posmenopausia**.

El ***objetivo principal*** de este estudio será evaluar si el efecto combinado de tres extractos vegetales a base de isoflavonas de soja, granada y granos del paraíso, mejoran la calidad de vida de mujeres perimenopaúsicas y menopaúsicas. Y, como ***objetivo secundario,*** evaluar la mejora en la calidad de vida por cada dimensión: menopausia y salud, psíquica, sexualidad y pareja, así como la no ganancia o la pérdida de peso tras la administración del extracto herbal combinado de soja, granos del paraíso y granada, ya que los principios activos de dichas plantas se asocian con propiedades antiinflamatorias, antioxidantes, y con una mejora del ánimo y una reducción del número de sofocos.

Se trata de un estudio denominado “doble ciego” en el que la mitad de las pacientes tomará el principio activo mientras que la otra mitad recibirá un placebo (sustancia inocua sin actividad biológica alguna). La asignación de las pacientes al tratamiento con principio activo o con placebo se realizará de forma aleatorizada (al azar) y ni el médico ni el paciente sabrán cuál es el tratamiento aplicado. La duración del estudio será de 8 semanas y se incluirán a 72 voluntarias de edades comprendidas entre los 45 y 55 años.

**¿Qué implica participar en el estudio?**

Ud. se comprometerá a asistir a las visitas con su ginecólogo. Habrá dos visitas, la del inicio (visita 1, V1) y la de seguimiento/finalización (visita 2, V2) y una llamada telefónica (visita 3, V3) con un tiempo estimado de visitas de 20-30 min para V1, de 15 min para V2 y 5 min para V3. Las visitas las programará su médico de la siguiente manera: semana 1 o visita de inicio, semana 9 o visita de seguimiento/finalización (día 56 ± 3) y llamada telefónica (día 63± 3). Las ventanas temporales para la visita 2 y 3 serán de 3 días, antes o después de la fecha señalada.

Ud. se comprometerá a seguir el tratamiento diario que consistirá en 2 cápsulas al día, una con el desayuno y otra con la cena, siempre con la comida.

En V1 Se le entregarán tres botes que contendrán 40 *pastillas* cada uno, haciendo un total de 120 pastillas que tendrá que tomar durante las 8 semanas siguientes a su entrega. Además, se le entregará un diario de seguimiento en formato tarjeta que podrá llevar con Ud. en su bolso y completar cuando sea necesario. En dicho diario tendrá que rellenar la información en relación al número de sofocos (de manera numérica) y a su estado anímico (con cruces). Tanto los botes con el contenido de pastillas que queden, así como el diario serán devueltos al médico en la visita V2.

Ud. se comprometerá a completar con el médico en sus visitas los cuestionarios de: Escala Cervantes (V1 y V2), cuestionario de salud y alimentación (V1 y V2), cuestionario de efectos adversos (V2) y cuestionario de medicación concomitante (V2) así como a la encuesta telefónica que se le realizará (V3).

Ud. se comprometerá a realizarse como prueba complementaria dos analíticas de sangre que será solicitado por su médico, uno al inicio del estudio y otro al finalizar el mismo, en las que se le pedirá un hemograma completo, un análisis bioquímico rutinario, sedimento de orina y determinación de proteinuria.

**¿Por qué ha sido elegida?**

Para que este estudio tenga éxito, los voluntarios serán seleccionados entre los que cumplan los criterios de inclusión y no tengan ningún tipo de problema médico.

**¿Tiene que participar?**

Depende de usted decidir si desea o no participar. Si usted decide participar se le pedirá que firme un formulario de consentimiento. Si decide participar, usted es libre de retirarse en cualquier momento y sin dar ninguna razón.

**¿Qué tengo que hacer?**

Tratar de seguir las indicaciones que recibirá por parte de su médico. NO es necesario que cambie sus hábitos alimenticios y deportivos diarios, pero se le aconsejará seguir una dieta equilibrada. Acudir a realizar las visitas y pruebas programadas. No tomar medicamentos que no hayan sido prescritos por su médico. Si necesita recibir algún tratamiento médico (por ejemplo, imagine que tiene una gripe) debe comunicar esa eventualidad. Se le facilitarán teléfonos de contacto y correos electrónicos para que pueda comunicarse con nosotros con facilidad.

**Beneficios y riesgos derivados de su participación en el estudio**

**¿Cuál es el medicamento, suplemento o procedimiento que se está probando?**

Este estudio evalúa el efecto de un suplemento nutricional, no se emplean medicamentos en esta investigación.

Se trata de un extracto natural a base de isoflavonas de soja, extracto de piel de granada y extracto de granos del paraíso. La soja es la principal fuente de isoflavonas, principio activo que comparte algunas similitudes con los estrógenos y ejerce efectos biológicos similares, reduciendo los efectos de su pérdida por la entrada en la menopausia, y que ha sido ampliamente usada para tratar los síntomas del climaterio.

La granada es una fruta con propiedades antioxidantes y antiinflamatorias. Además, se ha demostrado en estudios recientes que ayuda a prevenir la pérdida de masa ósea, regula los niveles de glucosa en sangre y actúa como prebiótico al mejorar la flora intestinal favoreciendo la absorción de las isoflavonas.

Los granos del paraíso son un condimento culinario, utilizado sólo o en combinación en la cocina de diversos países africanos. Diversas investigaciones le han atribuido propiedades beneficiosas para la salud, entre las que se cuentan sus efectos antioxidantes, antimicrobianos, y su posible utilidad como coadyuvante en la pérdida de peso.

La combinación de las isoflavonas de soja con los otros extractos de origen vegetal podría generar un efecto sinérgico que mejore la sintomatología del climaterio (flatos, sofocos, insomnio, etc.) y ayudar a controlar el peso.

**¿Cuál es el beneficio que podría obtener?**

Una reducción del número de sofocos diarios, un mayor descanso nocturno, así como un estado anímico mejorado, propiciando una vida más activa y un mayor control sobre la sintomatología depresiva o la ganancia de peso.

Es posible que usted no note ninguno de los efectos beneficiosos ya que es un estudio doble ciego, y ni usted ni su médico saben si está recibiendo el suplemento o placebo.

**¿Cuáles son los efectos secundarios de cualquier tratamiento o procedimientos recibidos al tomar parte de este estudio?**

No se han descrito efectos secundarios para los componentes por separado del suplemento a las dosis de la formulación.

Los componentes de este producto son considerados por la **Agencia Europea de la Alimentación** como alimentos o especias. No se han comercializado en conjunto previamente, aunque sí por separado como alimento o especias y no se han descrito efectos adversos derivados del consumo normal de los mismos. Sin embargo, el consumo de grandes cantidades de Granos del paraíso (7 veces más de lo habitual) se ha asociado a trastornos visuales y en estudios en ratas se observó una reducción en la ganancia de peso de las hembras en el último trimestre, por lo que no se aconseja su ingesta continua durante el embarazo.

**Tratamientos alternativos**

Un gran porcentaje de la población femenina no usa tratamiento para la sintomatología vasomotora, y la alivian siguiendo los hábitos saludables recomendados. Sin embargo, una de cada cuatro ve afectada su calidad de vida por lo que requiere de algún tipo de ayuda. Los tratamientos de uso habitual bajo prescripción médica son la **terapia hormonal sustitutoria** a base de estrógenos (TE) o combinada con progestágenos (TH) y los **inhibidores selectivos de la recaptación de serotonina (ISRS).**

**L**a **fitoterapia** es una alternativa a la terapia hormonal sustitutoria y consiste en el uso de plantas medicinales y sus derivados con fines terapéuticos. Los fitoestrógenos son los principios activos más habituales de uso entre los que se encuentra la genisteína, daidzeína y gliciteína, isoflavonas abundante en los vegetales como la soja (*Glycine max*) o en el trébol rojo (*Trifolium pratense*) por ejemplo.

Tanto la **Organización Mundial de la Salud** (OMS) como la **Sociedad Norteamericana de la Menopausia** recomiendan el uso de isoflavonas en dosis probadas clínicamente de 40-160 mg/día. La **Asociación Española para el Estudio de la Menopausia** (AEEM) aconseja que el preparado de fitoestrógenos tenga una dosis entre 40-80 mg/día con un mínimo de genisteína de 15 mg como terapia de uso alternativo para el tratamiento de la sintomatología vasomotora. A las recomendaciones de la AEEM se le une las recomendaciones de *La Agencia para la calidad e investigación de la asistencia médica* (Agency for Healthcare Research and Quality (AHRQ)) que resume que el rango de dosis consumidas de isoflavonas al día puede ser de 10-185 mg/dl con una media de 80 mg. A dosis elevadas de genisteína (60 mg/día durante 12 semanas) no se han observado efectos secundarios y sí una reducción significativa del número de sofocos. La adición de isoflavonas a los alimentos de uso diario podría reducir parte de la sintomatología del climaterio.

**Seguro**

En el improbable caso de una complicación tras el estudio o si deseara hacer una reclamación, podrá contactar en primer lugar con los médicos responsables de la ejecución del estudio. Si no hubiera un resultado satisfactorio, se trasladaría sus preocupaciones al Presidente del Comité de Ética.

Todos los médicos que participan en la investigación disponen de una cobertura personal de negligencia médica. Es decir, cualquier daño potencial está debidamente cubierto por pólizas de seguro.

**Confidencialidad**

**Si tomara parte del estudio, ¿Se mantendría la confidencialidad de mis datos?**

El tratamiento, la comunicación y la cesión de los datos de carácter personal de todos los sujetos participantes se ajustará a lo dispuesto en la Ley Orgánica 15/1999, de 13 de diciembre de protección de datos de carácter personal. De acuerdo a lo que establece la legislación mencionada, usted puede ejercer los derechos de acceso, modificación, oposición y cancelación de datos, para lo cual deberá dirigirse a su médico del estudio.

Los datos recogidos para el estudio estarán identificados mediante un código y solo el médico del estudio/colaboradores podrá relacionar dichos datos con usted y con su historia clínica. Por lo tanto, su identidad no será revelada a persona alguna salvo excepciones, en caso de urgencia médica o requerimiento legal.

Sólo se transmitirán a terceros y a otros países los datos recogidos para el estudio que en ningún caso contendrán información que le pueda identificar directamente, como nombre y apellidos, iniciales, dirección, nº de la seguridad social, etc. En el caso de que se produzca esta cesión, será para los mismos fines del estudio descrito y garantizando la confidencialidad como mínimo con el nivel de protección de la legislación vigente en nuestro país.

El acceso a su información personal quedará restringido al médico del estudio/colaboradores, autoridades sanitarias (Agencia Española del Medicamento y Productos Sanitarios), al Comité Ético de Investigación Clínica y personal autorizado por el promotor, cuando lo precisen para comprobar los datos y procedimientos del estudio, pero siempre manteniendo la confidencialidad de los mismos de acuerdo a la legislación vigente.

**¿Qué pasará con los resultados del estudio de investigación?**

Los datos recogidos durante el estudio podrían ser publicados en la literatura científica, lo que permitiría a otros profesionales utilizar dicha información. Usted no será identificado en ninguna publicación. Si desea estar informado de las publicaciones resultantes de este estudio, por favor, hágaselo saber a los investigadores del estudio.

**¿Quién financia la investigación?**

Esta investigación está financiada 100% por Nektium Pharma. SL, una empresa biotecnológica ubicada en Las Palmas de GC. La Clínica Baren aporta recursos propios de material y personal.

**¿Quién ha revisado el estudio?**

Este estudio ha sido revisado y aprobado por el Comité Ético de Investigación Humana de la ULPGC.

**¿Qué pasa con los datos genéticos y biológicos que se recojan?**

No está previsto recoger datos genéticos en este estudio. Las muestras biológicas serán tomadas por un laboratorio de análisis de referencia y los resultados evaluados por su médico.

**Cesión de datos personales y genéticos a terceros**

El equipo de investigación que lleva a cabo esta investigación no podrá ceder sus datos de carácter personal a terceros sin su autorización escrita. Sólo los investigadores que intervienen en este estudio tendrán acceso a sus datos personales.

**Otra información relevante**

Cualquier nueva información referente al producto utilizado en el estudio y que pueda afectar a su disposición para participar en el estudio, que se descubra durante su participación, le será comunicada por su médico lo antes posible.

Si usted decide retirar el consentimiento para participar en este estudio, ningún dato nuevo será añadido a la base de datos y, puede exigir la destrucción de todas las muestras identificables previamente retenidas para evitar la realización de nuevos análisis.

También debe saber que puede ser excluido del estudio si el promotor o los investigadores del estudio lo consideran oportuno, ya sea por motivos de seguridad, por cualquier acontecimiento adverso que se produzca por el producto en estudio o porque consideren que no está cumpliendo con los procedimientos establecidos. En cualquiera de los casos, usted recibirá una explicación adecuada del motivo que ha ocasionado su retirada del estudio.

Al firmar la hoja de consentimiento adjunta, se compromete a cumplir con los procedimientos del estudio que se le han expuesto.

Cuando acabe su participación recibirá el mejor tratamiento disponible y que su médico considere el más adecuado, pero es posible que no se le pueda seguir administrando el producto del estudio. Por lo tanto, ni el investigador ni el promotor adquieren compromiso alguno de mantener dicho tratamiento fuera de este estudio.

**CONFORMIDAD**

D/Dª. ____________________________________________________________

Manifiesto haber leído y comprendido la información facilitada en Hoja de Información para las voluntarias del estudio *Evaluación de un extracto herbal con isoflavonas (WH201601) sobre la sintomatología del climaterio (Cod del proyecto: PRO_WH_HCT_2016.01).* Así mismo, haber recibido respuesta satisfactoria a las preguntas que he planteado y que acepto voluntariamente participar en este estudio.

Fecha:_______________________

Firma:_________________________________Firma_________________________

Voluntario Investigador que informa

# ANEXO 2: Consentimiento informado (1 hojas)

**TÍTULO DEL ESTUDIO**:

Estudio del efecto de un suplemento nutricional con isoflavonas sobre la sintomatología del climaterio en mujeres sanas

**CÓDIGO DEL PROMOTOR:** PRO_WH_HCT_2016.01

**PROMOTOR:** Nektium Pharma SL

**INVESTIGADOR PRINCIPAL**: Dr. Miguel Barber (Clínica Baren), Dr. Ricardo Chirino (ULPGC)

**CENTRO:** Clínica Baren

**Responsable en el centro:** Dr. Miguel Barber

**Responsable Nektium Pharma** **SL**: Dra Laura López Ríos

Yo (nombre y apellidos) ................................................ y

DNI: ……………………………………………

He leído la hoja de información que se me ha entregado, he podido hacer preguntas sobre el estudio, he recibido suficiente información sobre el estudio y he hablado con: ...............................................................................(nombre del responsable investigador)

Comprendo que mi participación es voluntaria. Comprendo que puedo retirarme del estudio:

1º Cuando quiera

2º Sin tener que dar explicaciones.

3º Sin que esto repercuta en mis cuidados médicos.

Presto libremente mi conformidad para participar en este estudio y doy mi consentimiento para el acceso y utilización de mis datos en las condiciones detalladas en la hoja de información.

- SI
- NO

**Firma del paciente**:

Nombre:

**Firma del investigador**:

**Nombre:**

**Fecha**: **Fecha**:

Versión: Fecha:

# ANEXO 3: Cuestionario de salud (2 hojas)

**TÍTULO DEL ESTUDIO**:

Estudio del efecto de un suplemento nutricional con isoflavonas sobre la sintomatología del climaterio en mujeres sanas

**CÓDIGO DEL PROMOTOR:** PRO_WH_HCT_2016.01

**PROMOTOR:** Nektium Pharma SL

**INVESTIGADOR PRINCIPAL**: Dr. Miguel Barber (Clínica Baren), Dr. Ricardo Chirino (ULPGC)

**CENTRO:** Clínica Baren

**Responsable en el centro:** Dr. Miguel Barber

**Responsable Nektium Pharma** **SL**: Dra Laura López Ríos

**VARIABLES ANTROPOMÉTRICAS**

Edad: __________

Talla: __________

Peso:___________

Perímetro de cintura: _________________

Perímetro de cadera: _________________

Presión arterial: _____________________

Frecuencia cardiaca en reposo: __________________________

Temperatura sublingual: _______________________________

**CUESTIONARIO (marca la opción que proceda)**

**1**. Vamos a comenzar hablando de su salud. En los últimos doce meses ¿diría que su estado de salud ha sido muy bueno, bueno, regular, malo o muy malo? (Elija una opción)

- Muy bueno
- Bueno
- Regular
- Malo
- Muy malo

**2.** ¿Ha sido diagnosticado de alguna de las siguientes enfermedades o problemas de salud?

1. Cáncer ginecológico: mama, endometrio, cérvix.
2. Alergias alimentarias o intolerancias alimenticias: lácteos, huevos, frutos secos, chocolate…
3. Hipertensión arterial
4. Colesterol elevado
5. Diabetes (azúcar elevado)
6. Enfermedades del tiroides
7. Asma, bronquitis crónica o enfisema
8. Enfermedad del corazón
9. Úlcera de estómago
10. Bulimia
11. Anorexia Nerviosa
12. Depresión
13. Jaquecas, migrañas o dolores de cabeza
14. Hernias
15. Artrosis y problemas reumáticos
16. Osteoporosis

**3.** Indique si padece alguna otra enfermedad no recogida en la lista anterior

----------------------------------------------------------------------------------------------------------------------------------------------------------------------------------------------------------------------------------------

**4.-** En caso de que las preguntas 2 y/o 3 sean afirmativas ¿está ahora en tratamiento para dicha enfermedad? En caso afirmativo ¿con qué?

-----------------------------------------------------------------------------------------------------------------------------

-----------------------------------------------------------------------------------------------------------------------------

---------------------------------------------------------------

**5.** ¿Hay antecedentes en su familia de cáncer ginecológico (mama, endometrio, cérvix)?

□ SÍ □NO

**6**. ¿Cuándo tuvo la última menstruación? ……………………………………………………

**7.** ¿Cada cuánto tiempo ha tenido las últimas menstruaciones? ……………………………….

**8.** ¿Las últimas menstruaciones son: más abundantes de lo normal o menos abundantes de lo normal? ………………………………………

**9.** ¿Está tomando algún tratamiento hormonal (píldoras anticonceptivas, tratamiento hormonal sustitutorio, tratamiento de tiroides, etc)? En caso afirmativo, ¿cuál?

□SÍ □NO ………………………………………………………………………………………

**10.** ¿Toma algún otro medicamento de manera habitual? En caso afirmativo, ¿cuál?

□SÍ □NO …………………………………………………………………………………….

**11**. ¿Es fumadora?

□SÍ. □Nº Cigarros/día:………. □Ocasional, sólo cuando salgo □NO

**12.-** ¿Práctica algún deporte? En caso afirmativo indique qué tipo de deporte, con cuanta frecuencia y duración

…………………………………………………………………………………………………………..

………………………………………………………………………………………………………….

***Nota:*** Las preguntas 2a, 2b, 2g y 9, si son positivas son criterios de exclusión directos.

# ANEXO 4: Recomendaciones dietéticas (2 hojas)

**TÍTULO DEL ESTUDIO**:

Estudio del efecto de un suplemento nutricional con isoflavonas sobre la sintomatología del climaterio en mujeres sanas

**CÓDIGO DEL PROMOTOR:** PRO_WH_HCT_2016.01

**PROMOTOR:** Nektium Pharma SL

**INVESTIGADOR PRINCIPAL**: Dr. Miguel Barber (Clínica Baren), Dr. Ricardo Chirino (ULPGC)

**CENTRO:** Clínica Baren

**Responsable en el centro:** Dr. Miguel Barber

**Responsable Nektium Pharma** **SL**: Dra Laura López Ríos

Mantener una dieta equilibrada: incluyendo todos los grupos de alimentos para conseguir los nutrientes necesarios.

Comer de manera moderada en cada comida y realizar 5 comidas al día, no dejando que lleguemos a las comidas principales con mucha hambre.

Hidratación, agua: consumir un mínimo de 1,5l de agua al día (6-8 vasos).

Limitar el consumo: Harinas refinadas, azúcar blanco, bollería, pan de molde y zumos industriales, así como refrescos

Frecuencias/raciones de alimentos por grupo:

| **Alimento** | **Veces** | **Ración (gr)** | **Ración** |
| --- | --- | --- | --- |
| **Hidratos de carbono** | 4-6 rac/día |  |  |
| Pasta/arroz |  | 60-80 gr | Plato normal |
| Pan |  | 40-60 gr | 3-4 rebanadaso un panecillo |
| Papas |  | 150-200gr | 1 grande o 2 pequeñas |
| **Verduras y hortalizas** | Más de 2 rac/día | 150-200 gr | Plato normal |
| **Frutas** | Más de 3 rac/día | 120-200 gr | 1 pieza mediana, 1 taza de cerezas, fresas, dos rodajas de melón |
| **Legumbre** | 2-4 rac/sem | 60-80 gr | Plato normal |
| **Pescado** | 3-4 ra/sem | 125-150 gr | 1 filete |
| **Proteínas animales** | 3-4 rac/sem |  |  |
| Carne magra |  | 100-120 gr | 1 filete pequeño |
| aves |  | 100-120 gr | ¼ pollo |
| huevos |  | 100-120 gr | 1-2 huevos |
| **Leches y derivados** | 2-4 rac/día |  |  |
| Leche |  | 200-250 ml | 1 taza |
| Yogur |  | 200-250 gr | 1-2 yogur |
| Queso curado |  | 40-60 gr | 2-3 lonchas |
| Queso fresco |  | 80-1250 gr | 1 porcion |
| **Frutos secos** | 3-7 rac/sem | 20-30 gr | 1 puñado |

**Embutidos y carne grasa:** consumo ocasional y moderado, **Dulces, snacks y refrescos:** consumo ocasional y moderado, **Mantequilla, margarina o bollería:** consumo ocasional y moderado, **Cerveza o vino/sidra:** consumo ocasional y moderado.

# Anexo 5: Cuestionario de alimentación (consumo de soja) (1 hoja)

**TÍTULO DEL ESTUDIO**:

Estudio del efecto de un suplemento nutricional con isoflavonas sobre la sintomatología del climaterio en mujeres sanas

**CÓDIGO DEL PROMOTOR:** PRO_WH_HCT_2016.01

**PROMOTOR:** Nektium Pharma SL

**INVESTIGADOR PRINCIPAL**: Dr. Miguel Barber (Clínica Baren), Dr. Ricardo Chirino (ULPGC)

**CENTRO:** Clínica Baren

**Responsable en el centro:** Dr. Miguel Barber

**Responsable Nektium Pharma** **SL**: Dra Laura López Ríos

1. ¿Sigue una alimentación vegetariana?

□SÍ □NO

1. ¿Consume de manera habitual y conscientemente, alimentos enriquecidos con soja (lácteos, carne de soja, brotes de soja, etc.)?

□SÍ □NO

**3.-** ¿Es alérgica a alguno de los siguientes alimentos?

Soja o derivados de soja: □Sí □ NO

Pimienta (especia): □ Sí □ NO

Jengibre: □Sí □ NO

Granada: □ Sí □ NO

**4.** De la siguiente lista de alimentos indique con qué frecuencia los consume

Varias veces al día (1). Varias veces a la semana (2). Varias veces al mes (3). De manera poco habitual (4). Nunca (5)

-Leche de Soja……………………………………………….

 -Tofu……………………………………………………….

 -Yogures/postres/helados/batidos de (o con) soja………….

 -Panes/repostería de (o con) harinas de Soja……………….

- Hamburguesas de soja…………………………………….

- Aislado de proteínas de Soja ………………………………

- Salsa de soja ………………………………………………

- Granos de Soja sin procesar ……………………………….

-Otro alimento que considere que puede derivar/contener Soja …………………………………

# Anexo 6: Escala Cervantes (2 hojas)

**TÍTULO DEL ESTUDIO**:

Estudio del efecto de un suplemento nutricional con isoflavonas sobre la sintomatología del climaterio en mujeres sanas

**CÓDIGO DEL PROMOTOR**: PRO_WW_HCT_2016.16

**PROMOTOR:** Nektium Pharma SL

**INVESTIGADOR PRINCIPAL**: Dr. Miguel Barber (Clínica Baren), Dr. Ricardo Chirino (ULPGC)

**CENTRO:** Clínica Baren

**Responsable en el centro: Dr. Miguel Barber**

**Responsable Nektium Pharma SL:** Dra Laura López Ríos

**Escala Cervantes de calidad de vida relacionada con la Salud** (Palacios 2004)

Nombre y apellido (iniciales): ___________________________________________________________

Nivel de estudios: 🞎 Sin estudios 🞎 Primarios 🞎 Secundarios 🞎 Universitarios

Fecha de nacimiento: ____________________________ Fecha actual: __________________________

Por favor, **lea atentamente cada una de las preguntas que vienen a continuación**. Comprobará que al lado del 0 y el 5 aparecen unas palabras que representan las dos formas opuestas de responder a la pregunta. Además, entre el 0 y el 5 figuran 4 casillas numeradas del 1 al 4. Responda a las preguntas y marque con una X la casilla que considere más adecuada según el grado de acuerdo entre lo que usted piensa y siente y las respuestas que se proponen. Es decir, si está totalmente de acuerdo marque el 5 y si está totalmente en desacuerdo marque el 0. Si no está totalmente de acuerdo o en desacuerdo utilice las casillas intermedias.

No piense demasiado las respuestas ni emplee mucho tiempo en contestarlas. Recuerde que no hay respuestas buenas o malas, ni respuestas con trampa, y todas deben responderse con sinceridad. Quizá considere que algunas preguntas son demasiado personales; no se preocupe, recuerde que este cuestionario es totalmente anónimo y confidencial de cara al estudio.

| **Preguntas** | **Valoración** | | | | | | | |
| --- | --- | --- | --- | --- | --- | --- | --- | --- |
| **1. Durante el día noto que la cabeza me va doliendo cada vez más** | Nunca | 0 | 1 | 2 | 3 | 4 | 5 | Todos los días |
| **2. No puedo más de lo nerviosa que estoy** | Nunca | 0 | 1 | 2 | 3 | 4 | 5 | Constantemente |
| **3. Noto mucho calor de repente** | Nunca | 0 | 1 | 2 | 3 | 4 | 5 | En todo momento |
| **4. Mi interés por el sexo se mantiene como siempre** | Mucho menos | 0 | 1 | 2 | 3 | 4 | 5 | Igual o más |
| **5. No consigo dormir las horas necesarias** | Nunca me ocurre | 0 | 1 | 2 | 3 | 4 | 5 | Constantemente |
| **6. Todo me aburre, incluso las cosas que antes me divertían** | No es cierto | 0 | 1 | 2 | 3 | 4 | 5 | Cierto |
| **7. Noto hormigueos en las manos y/o los pies** | No, en absoluto | 0 | 1 | 2 | 3 | 4 | 5 | Insoportable |
| **8. Me considero feliz en mi relación de pareja** | Nada | 0 | 1 | 2 | 3 | 4 | 5 | Completamente |
| **9. De pronto noto que empiezo a sudar sin que haya hecho ningún esfuerzo** | Nunca | 0 | 1 | 2 | 3 | 4 | 5 | Constantemente |
| **10. He perdido la capacidad de relajarme** | No, en absoluto | 0 | 1 | 2 | 3 | 4 | 5 | Completamente |
| **11. Aunque duermo, no consigo descansar** | Nunca me ocurre | 0 | 1 | 2 | 3 | 4 | 5 | Constantemente |
| **12. Noto como si las cosas me dieran vueltas** | Nada | 0 | 1 | 2 | 3 | 4 | 5 | Mucho |
| **13. Mi papel como esposa o pareja es...** | Nada importante | 0 | 1 | 2 | 3 | 4 | 5 | Muy importante |
| **14. Creo que retengo líquido, porque estoy hinchada** | No, como siempre | 0 | 1 | 2 | 3 | 4 | 5 | Sí, mucho más |
| **15. Estoy satisfecha con mis relaciones sexuales** | Nada | 0 | 1 | 2 | 3 | 4 | 5 | Completamente |
| **16. Noto que los músculos o las articulaciones me duelen** | No, en absoluto | 0 | 1 | 2 | 3 | 4 | 5 | Dolor insoportable |
| **17. Creo que los demás estarían mejor sin mí** | No, en absoluto | 0 | 1 | 2 | 3 | 4 | 5 | Cierto |
| **18. Me da miedo hacer esfuerzos porque se me escapa la orina** | No, en absoluto | 0 | 1 | 2 | 3 | 4 | 5 | Mucho |
| **19. Desde que me levanto me encuentro cansada** | Nada | 0 | 1 | 2 | 3 | 4 | 5 | Mucho |
| **20. Tengo tan buena salud como cualquier persona a mi edad** | No, en absoluto | 0 | 1 | 2 | 3 | 4 | 5 | Igual o mejor |
| **21. Tengo la sensación de que no sirvo para nada** | Nunca | 0 | 1 | 2 | 3 | 4 | 5 | En todo momento |
| **22. Tengo relaciones sexuales tan a menudo como antes** | Mucho menos | 0 | 1 | 2 | 3 | 4 | 5 | Igual o más |
| **23. Noto que el corazón me late muy deprisa y sin control** | Nada | 0 | 1 | 2 | 3 | 4 | 5 | Mucho |
| **24. A veces pienso que no me importaría estar muerta** | Nunca | 0 | 1 | 2 | 3 | 4 | 5 | Constantemente |
| **25. Mi salud me causa problemas con los trabajos domésticos** | En absoluto | 0 | 1 | 2 | 3 | 4 | 5 | Constantemente |
| **26. En mi relación de pareja me siento tratada de igual a igual** | Nunca | 0 | 1 | 2 | 3 | 4 | 5 | Siempre |
| **27. Siento picor en la vagina, como si estuviera demasiado seca** | Nada | 0 | 1 | 2 | 3 | 4 | 5 | Mucho |
| **28. Me siento vacía** | Nunca | 0 | 1 | 2 | 3 | 4 | 5 | Siempre |
| **29. Noto sofocaciones** | Nunca | 0 | 1 | 2 | 3 | 4 | 5 | En todo momento |
| **30. En mi vida el sexo es...** | Nada importante | 0 | 1 | 2 | 3 | 4 | 5 | Extremadamente importante |
| **31. He notado que tengo más sequedad de piel** | No, como siempre | 0 | 1 | 2 | 3 | 4 | 5 | Sí, mucho más |

# ANEXO 7: Cuestionario de seguimiento/finalización (2 hojas)

**TÍTULO DEL ESTUDIO**:

Estudio del efecto de un suplemento nutricional con isoflavonas sobre la sintomatología del climaterio en mujeres sanas

**CÓDIGO DEL PROMOTOR**: PRO_WW_HCT_2016.16

**PROMOTOR:** Nektium Pharma SL

**INVESTIGADOR PRINCIPAL**: Dr. Miguel Barber (Clínica Baren), Dr. Ricardo Chirino (ULPGC)

**CENTRO:** Clínica Baren

**Responsable en el centro: Dr. Miguel Barber**

**Responsable Nektium Pharma SL:** Dra Laura López Ríos

**VARIABLES ANTROPOMÉTRICAS**

- Peso: __________________________
- Presión arterial: __________________
- Perímetro de cintura: ______________
- Perímetro de cadera: _______________
- Presión arterial: _____________________
- Frecuencia cardiaca en reposo: __________________________
- Temperatura sublingual: _______________________________

**CUESTIONARIO** (marca la opción que proceda)

**1.** ¿Cómo ha sido su estado de salud estas semanas de manera general?

- Muy bueno
- Bueno
- Regular
- Malo
- Muy malo

**2.** ¿Ha tenido la menstruación en las últimas semanas? ………………………………………

**3.** ¿Ha tenido más de una menstruación en las últimas semanas?, ¿cuántas?

**4.** En caso afirmativo ¿cuánto tiempo han durado? …………………………………………….

**5.** ¿Ha tomado algún tratamiento alternativo para tratar los síntomas del climaterio (píldoras anticonceptivas, tratamiento hormonal sustitutorio, etc)? En caso afirmativo, ¿Cuál?

□SÍ □NO ………………………………………………………………………………….

**6.-** ¿Ha mantenido su rutina de alimentación?

□ SÍ □ NO, he seguido la pauta que nos han dado □ NO

**7.-** ¿Ha seguido su rutina de deporte?

□SÍ □NO, he practicado más □ NO, he practicado menos

**8.-** Posibles efectos secundarios:

¿Ha estado enferma en las últimas semanas o ha acudido al médico por alguna afección? En caso afirmativo ¿Por qué?

*El médico deberá indicar si creo que puede haber alguna relación con el tratamiento. Indicando si la relación es:* ***Probable****, en caso de que considere que haya una relación entre el tratamiento y la dolencia (****3****),* ***Posible,*** *en caso de que no tenga claro que la relación entre el tratamiento y la dolencia sea directa (****2****) o si* ***no cree que haya relación*** *(****1****).*

**9.-** Medicación concomitante. ¿Ha tomado algún tipo de medicación desde que inició el tratamiento? En caso afirmativo: ¿Qué medicación?, ¿Para qué? Y ¿Cuánto tiempo estuvo tomándola?

**VALORACIÓN DEL MÉDICO**

En función de las respuestas dadas por esta participante considero que podría estar recibiendo:

A: Placebo 🞎

B: Producto 🞎

Responsable C. Baren:

Fecha

# ANEXO 8: Cuestionario telefónico (1 hoja)

**TÍTULO DEL ESTUDIO**:

Estudio del efecto de un suplemento nutricional con isoflavonas sobre la sintomatología del climaterio en mujeres sanas

**CÓDIGO DEL PROMOTOR**: PRO_WW_HCT_2016.16

**PROMOTOR:** Nektium Pharma SL

**INVESTIGADOR PRINCIPAL**: Dr. Miguel Barber (Clínica Baren), Dr. Ricardo Chirino (ULPGC)

**CENTRO:** Clínica Baren

**Responsable en el centro: Dr. Miguel Barber**

**Responsable Nektium Pharma SL:** Dra Laura López Ríos

**1.** ¿Ha tenido la menstruación en la última semana?…………………………………………

**2.** ¿Ha dormido bien en la última semana?

Sí 🞎 No 🞎 Como siempre 🞎

**3**. ¿Notado sofocos en la última semana? En caso afirmativo, ¿Cuántos? _____________________

4.- Cómo ha sido su estado de salud estas semanas de manera general (elija una opción)

- Muy bueno
- Bueno
- Regular
- Malo
- Muy malo

**5**.- ¿Cómo ha sido su estado de ánimo en la última semana?

- Normal:
- Contenta:
- Sensible:
- Irritada:
- Deprimida:
- Cansada:
- Otro: _________________________________________

**6**. Posibles efectos secundarios:

¿Ha estado enferma en la última semana o ha acudido al médico por alguna afección? En caso afirmativo ¿Por qué?

*El médico deberá indicar si cree que puede haber alguna relación con el tratamiento, Indicando si la relación es:* ***Probable****, en caso de que considere que haya una relación entre el tratamiento y la dolencia (****3****),* ***Posible,*** *en caso de que no tenga claro que la relación entre el tratamiento y la dolencia sea directa (****2****) o si N****o cree que haya relación*** *(****1****).*

7. Medicación concomitante. ¿Ha tomado algún tipo de medicación? En caso afirmativo: ¿Qué medicación?, ¿Para qué? Y ¿Cuánto tiempo ha estado tomándola?

Responsable C. Baren:

Fecha

# ANEXO 9: Diarios de seguimiento (2 hojas)

**TÍTULO DEL ESTUDIO**:

Estudio del efecto de un suplemento nutricional con isoflavonas sobre la sintomatología del climaterio en mujeres sanas

**CÓDIGO DEL PROMOTOR**: PRO_WW_HCT_2016.16

**PROMOTOR:** Nektium Pharma SL

**INVESTIGADOR PRINCIPAL**: Dr. Miguel Barber (Clínica Baren), Dr. Ricardo Chirino (ULPGC)

**CENTRO:** Clínica Baren

**Responsable en el centro: Dr. Miguel Barber**

**Responsable Nektium Pharma SL:** Dra Laura López Ríos

**PORTADA:**

- TÍTULO: Diario del participante…………NÚMERO: ……….
- PATROCINADORES: Clínica Baren + Nektium
- FECHA DE ENTREGA: ……
- FECHA DE RECOGIDA: ………………………….

**1ª HOJA:**

- INSTRUCIONES DE USO: El diario debe de ser completado diariamente con la información que requiere. Es aconsejable llevarlo con Uds. en el bolso o tenerlo en un lugar al que le sea fácil acceder como la mesa de noche. La información que debe de completar es:
  - *Número de sofocos al día*: Marque con una cruz cada vez que sienta uno o complételo al final del día
  - *Estado de ánimo:* Marque con un número el estado de ánimo más común a lo largo del día siguiendo la leyenda que se adjunta. Cómo mucho indicar dos estados de ánimo.
- DUDAS:
- *¿Qué pasa si un día me olvido de tomar el tratamiento?:*
  - No se preocupe, tómese la siguiente dosis, el tratamiento se puede prolongar un día más, pero anote en observaciones que día no se lo tomo.
- *¿Puede sentarme mal?:*
  - El tratamiento es un producto natural considerado como alimento que no debería de sentarle mal. En caso de que no se sienta bien póngase en contacto con su médico
- *El médico me ha recetado un medicamento ¿afecto al tratamiento?*
  - No tendría por qué, Pero sí debe de anotar lo que se toma e indicárselo al personal médico de la clínica Baren
- DE ESTADO DE ÁNIMO:
- 1. Irritada
- 2. Confusa
- 3. Triste/deprimida
- 4. Cansada
- 5. Sensible
- 6. Norma
- 7. Contenta

**PENÚLTIMA HOJA**: Observaciones e incidencias

**ÚLTIMA HOJA**: Información de contacto
