# Supplementary material for: Influence of a new botanical combination on quality of life in menopausal Spanish women: Results of a randomized, placebo-controlled pilot study
Source: PLoS One. 2021 Jul 21;16(7):e0255015. doi: 10.1371/journal.pone.0255015 (PMC8294509; doi:10.1371/journal.pone.0255015)
Supplement: S3 File — (DOCX) [file pone.0255015.s003.docx]

Annexes

Study of the effect of a nutritional supplement with isoflavones on the symptoms of climacteric in healthy women

**Proyect Code: PRO_WH_HCT_2016.01**

INDEX

[I. ANNEXE 1: Information to the participant (7 sheets) 3](#_Toc473728091)

[II. ANNEXE 2: Informed consent (1 sheets) 10](#_Toc473728092)

[III.ANNEXE 3: Health questionnaire (2 sheets) 11](#_Toc473728093)

[IV. ANNEXE 4: Dietary recommendations (2 sheets) 13](#_Toc473728094)

[V.ANNEXE 5: Diet questionnaire (soy consumption) (1 sheet) 15](#_Toc473728095)

[VI. ANNEXE 6: Cervantes Scale (2 sheets) 16](#_Toc473728096)

[VII. ANNEXE 7: Follow-up / Completion Questionnaire (2 sheets) 18](#_Toc473728097)

[VIII. ANNEXE 8: Telephone questionnaire (1 sheet) 20](#_Toc473728098)

[IX. ANNEXE 9: Tracking personal diaries (2 sheets) 22](#_Toc473728099)

# ANNEXE 1: Information to the participant (7 sheets)

**TITLE OF THE STUDY:**

Study of the effect of a nutritional supplement with isoflavones on the symptoms of climacteric in healthy women

**PROMOTER CODE:** PRO_WH_HCT_2016.01

**PROMOTER:** Nektium Pharma SL

**PRINCIPAL INVESTIGATOR:** Dr. Miguel Barber (Baren Clinic), Dr. Ricardo Chirino (ULPGC)

**CENTER:** Baren Clinic

**Person in charge at the center:** Dr. Miguel Barber

**Head of Nektium Pharma SL:** Dra Laura López Ríos

**Introduction**

We write to you about a research study in which you are invited to participate. The product to be tested is a nutritional supplement, and the study has been approved by the Human Research Ethics Committee of the University of Las Palmas de Gran Canaria (ULPGC). Our intention is only that you receive the correct and sufficient information so that you can evaluate and judge whether or not you want to participate in this study. To do this, read this information sheet carefully and we will clarify any doubts that may arise. In addition, you can consult with the people you consider appropriate

**Voluntary engagement**

You should know that your participation in this study is voluntary and that you can decide not to participate or change your decision and withdraw your consent at any time, without altering your relationship with your doctor or causing any harm to your treatment.

**Study overview**

Both men and women experience both physical and mental decline over the years. In the case of women, the loss of estrogens is associated with a specific type of symptoms known as climacteric, which can reduce their quality of life. The Climacteric is known as the transition period that lasts for years, both before (perimenopause), during (menopause) and after menopause (postmenopause), as a consequence of ovarian exhaustion, associated with a decrease in its ability to produce hormones, follicles and oocytes. The most common age range is usually from 45 to 55 years, with the mean age of menopause in our country being 51 years (National Statistics Institute, 2011). It is a period of involution in which the gradual loss of estrogens occurs and is accompanied by a series of physical and emotional manifestations related to biological and social changes (hot flashes, weight gain, insomnia, irritability, decreased libido, etc. ). All manifestations are not common to all women who are going through this stage, but depend on each woman

Perimenopause extends from the moment the first menstrual cycle changes appear until the year following the definitive cessation of menstruation. It begins with an increase in vaginal bleeding (bleeding for more than 7 days), followed by a spacing between menses (amenorrhea of more than 60 days) and last 12 months in a row without menstruation. The duration can vary between two and five years. Gradual suppression of ovarian activity occurs, both in the quantity and quality of oocytes, making it a period of low fertility with changes in estrogen levels that tend to disappear. Menopause is the period in a woman's life when estrogen is lacking due to the cessation of ovarian activity and is therefore no longer fertile. It is considered to start after a full year without menstruation. After that year there is talk of postmenopause.

The main objective of this study will be to evaluate whether the combined effect of three vegetable extracts based on soy isoflavones, pomegranate and grains of paradise, improve the quality of life of perimenopausal and menopausal women. And, as a secondary objective, to evaluate the improvement in the quality of life for each dimension: menopause and health, psychic, sexuality and partner, as well as the no gain or loss of weight after the administration of the combined herbal extract of soybeans, paradise and pomegranate, since the active principles of these plants are associated with anti-inflammatory, antioxidant properties, and with an improvement in mood and a reduction in the number of hot flashes.

This is a study called "double blind" in which half of the patients will take the active ingredient, while the other half will receive a placebo (a harmless substance with no biological activity). The assignment of the patients to the treatment with active ingredient or with placebo will be carried out in a randomized (random) way and neither the doctor nor the patient will know which treatment is applied. The duration of the study will be 8 weeks and 72 volunteers of ages between 45 and 55 will be included.

**What does participating in the study involve?**

You will commit to attend visits with your gynecologist. There will be two visits, the start visit (visit 1, V1) and the follow-up / end visit (visit 2, V2) and a phone call (visit 3, V3) with an estimated visit time of 20-30 min for V1, from 15 min for V2 and 5 min for V3. The visits will be scheduled by your doctor as follows: week 1 or start visit, week 9 or follow-up / end visit (day 56 ± 3) and phone call (day 63 ± 3). The temporary windows for visit 2 and 3 will be 3 days, before or after the date indicated.

You will commit to follow the daily treatment that will consist of 2 capsules a day, one with breakfast and the other with dinner, always with lunch.

In V1 you will be given three cans that will contain 40 pills each, making a total of 120 pills that you will have to take during the 8 weeks following your delivery. In addition, you will be given a follow-up diary in card format that you can carry with you in your bag and complete when necessary. In this newspaper, you will have to fill in the information regarding the number of hot flashes (numerically) and your mood (with crosses). Both the cans with the contents of the pills that remain, as well as the diary will be returned to the doctor at visit V2.

You will commit to completing with your doctor the questionnaires of: Cervantes Scale (V1 and V2), health and nutrition questionnaire (V1 and V2), questionnaire of adverse effects (V2) and questionnaire of concomitant medication (V2) as well as the telephone survey that will be carried out (V3).

You will undertake to carry out as a complementary test two blood tests that will be requested by your doctor, one at the beginning of the study and the other at the end of the study, in which you will be asked for a complete blood count, a routine biochemical analysis, urine sediment and determination of proteinuria.

**Why has it been chosen?**

For this study to be successful, volunteers will be selected from those who meet the inclusion criteria and do not have any type of medical problem.

**Do you have to participate?**

It is up to you to decide whether or not you want to participate. If you decide to participate you will be asked to sign a consent form. If you decide to participate, you are free to withdraw at any time and without giving any reason.

**That I have to do?** Try to follow the directions you will receive from your doctor. You do NOT need to change your daily eating and sports habits, but you will be advised to follow a balanced diet. Attend the scheduled visits and tests. Do not take medications that have not been prescribed by your doctor. If you need to receive any medical treatment (for example, suppose you have the flu) you should communicate this eventuality. Contact numbers and emails will be provided so that you can easily contact us.

**Benefits and risks derived from your participation in the study**

**What is the medication, supplement, or procedure being tested?**

This study evaluates the effect of a nutritional supplement, no drugs are used in this research. It is a natural extract based on soy isoflavones, pomegranate skin extract and grain of paradise extract. Soy is the main source of isoflavones, an active ingredient that shares some similarities with estrogens and exerts similar biological effects, reducing the effects of its loss due to menopause, and which has been widely used to treat climacteric symptoms.

Pomegranate is a fruit with antioxidant and anti-inflammatory properties. In addition, it has been shown in recent studies that it helps prevent bone loss, regulates blood glucose levels and acts as a prebiotic by improving the intestinal flora, favoring the absorption of isoflavones.

The grains of paradise are a culinary condiment, used alone or in combination in the cuisine of various African countries. Various investigations have attributed beneficial health properties, including its antioxidant, antimicrobial effects, and its possible usefulness as an aid in weight loss.

The combination of soy isoflavones with the other extracts of plant origin could generate a synergistic effect that improves the symptoms of the climacteric (flatus, hot flashes, insomnia, etc.) and help control weight.

**What is the benefit you could get?**

A reduction in the number of daily hot flashes, a greater night's rest, as well as an improved state of mind, promoting a more active life and greater control over depressive symptoms or weight gain.

You may not notice any of the beneficial effects since it is a double-blind study, and neither you nor your doctor know if you are receiving the supplement or placebo.

**What are the side effects of any treatment or procedures received from taking part in this study?**

No side effects have been described for the separate components of the supplement at the formulation doses. The components of this product are considered by the European Food Agency as food or spices. They have not been previously marketed together, although they have been separately sold as food or spices, and no adverse effects derived from their normal consumption have been described. However, the consumption of large amounts of Grains of paradise (7 times more than usual) has been associated with visual disturbances and in studies in rats a reduction in the weight gain of females was observed in the last trimester, therefore that its continuous intake during pregnancy is not recommended.

**Alternative treatments**

A large percentage of the female population does not use treatment for vasomotor symptoms, and they alleviate it by following the recommended healthy habits. However, one in four affects their quality of life, which requires some type of help. Commonly prescribed treatments are estrogen-based hormone replacement therapy (TE) or combined with progestogens (HT) and selective serotonin reuptake inhibitors (SSRIs).

Phytotherapy is an alternative to hormone replacement therapy and consists of the use of medicinal plants and their derivatives for therapeutic purposes. Phytoestrogens are the most common active ingredients in use, including genistein, daidzein and glycitein, abundant isoflavones in vegetables such as soybeans (Glycine max) or red clover (Trifolium pratense) for example.

Both the World Health Organization (WHO) and the North American Menopause Society recommend the use of isoflavones in clinically proven doses of 40-160 mg/day. The Spanish Association for the Study of Menopause (AEEM) advises that the preparation of phytoestrogens have a dose between 40-80 mg/day with a minimum of 15 mg of genistein as alternative therapy for the treatment of vasomotor symptoms. The AEEM recommendations are joined by the recommendations of the Agency for Healthcare Research and Quality (AHRQ) which summarizes that the range of consumed doses of isoflavones per day can be 10 -185 mg/dl with an average of 80 mg. At high doses of genistein (60 mg/day for 12 weeks) no side effects were observed and a significant reduction in the number of hot flashes was observed. Adding isoflavones to everyday foods may reduce some of the symptoms of climacteric.

**Study insurance**

In the unlikely event of a post-study complication or if you wish to make a claim, you may first contact the physicians responsible for conducting the study. If there is no satisfactory result, your concerns will be transferred to the Chairman of the Ethics Committee.

All physicians participating in the investigation have personal medical malpractice coverage. In other words, any potential damage is duly covered by insurance policies.

**confidentiality**

**If I took part in the study, would my data be kept confidential?**

The treatment, communication and transfer of personal data of all participating subjects will comply with the provisions of Organic Law 15/1999, of December 13, on the protection of personal data. In accordance with the provisions of the aforementioned legislation, you can exercise the rights of access, modification, opposition and cancellation of data, for which you must contact your study doctor.

The data collected for the study will be identified by a code and only the study doctor / collaborators will be able to relate these data to you and your medical history. Therefore, your identity will not be revealed to any person except for exceptions, in case of medical emergency or legal requirement.

Only the data collected for the study will be transmitted to third parties and to other countries, which in no case will contain information that can directly identify you, such as name and surname, initials, address, social security number, etc. In the event that this transfer occurs, it will be for the same purposes of the study described and guaranteeing confidentiality at least with the level of protection of the legislation in force in our country.

Access to your personal information will be restricted to the study doctor / collaborators, health authorities (Spanish Agency for Medicines and Health Products), the Clinical Research Ethics Committee and personnel authorized by the promoter, when they need it to check the data and procedures of the study, but always maintaining their confidentiality according to current legislation.

**What will happen to the results of the research study?**

The data collected during the study could be published in the scientific literature, which would allow other professionals to use this information. You will not be identified in any publication. If you would like to be informed of the publications resulting from this study, please let the study investigators know.

**Who funds the research?**

This research is 100% funded by Nektium Pharma. SL, a biotechnology company located in Las Palmas de GC. The Baren Clinic contributes its own material and personnel resources.

**Who has reviewed the study?**

This study has been reviewed and approved by the ULPGC Human Research Ethics Committee.

**What happens to the genetic and biological data that is collected?**

It is not planned to collect genetic data in this study. The biological samples will be taken by a reference analysis laboratory and the results evaluated by your doctor.

**Transfer of personal and genetic data to third parties**

The research team that carries out this research may not transfer your personal data to third parties without your written authorization. Only the researchers involved in this study will have access to your personal data.

**Additional relevant information**

Any new information regarding the product used in the study that may affect your willingness to participate in the study, that is discovered during your participation, will be communicated to you by your doctor as soon as possible.

If you decide to withdraw consent to participate in this study, no new data will be added to the database, and you may require the destruction of all identifiable samples previously retained to avoid further analysis.

You should also know that you may be excluded from the study if the study sponsor or investigators consider it appropriate, either for safety reasons, for any adverse event that occurs due to the product under study or because they consider that you are not complying with the procedures. established. In either case, you will receive an adequate explanation of the reason for your withdrawal from the study.

By signing the attached consent form, you agree to comply with the study procedures outlined to you.

When your participation ends, you will receive the best treatment available and that your doctor deems most appropriate, but you may not be able to continue receiving the study product. Therefore, neither the researcher nor the promoter make any commitment to keep this treatment out of this study.

**COMPLIANCE**

Mr./Ms. ____________________________________________________________

I declare that I have read and understood the information provided in the Information Sheet for the study volunteers Evaluation of an herbal extract with isoflavones (WH201601) on the symptoms of climacteric (Project code: PRO_WH_HCT_2016.01). Likewise, having received satisfactory answers to the questions that I have asked and that I voluntarily agree to participate in this study.

Date:_______________________

Signature:___________________________Signature:______________________________

Volunteer The reporting researcher

# ANNEXE 2: Inform consent (1 Sheet)

**TITLE OF THE STUDY:**

Study of the effect of a nutritional supplement with isoflavones on the symptoms of climacteric in healthy women

**PROMOTER CODE:** PRO_WH_HCT_2016.01

**PROMOTER:** Nektium Pharma SL

**PRINCIPAL INVESTIGATOR:** Dr. Miguel Barber (Baren Clinic), Dr. Ricardo Chirino (ULPGC)

**CENTER:** Baren Clinic

**Person in charge at the center:** Dr. Miguel Barber

**Head of Nektium Pharma SL:** Dra Laura López Ríos

I (name and surname) ................................................

DNI number: ……………………………………………

I have read the information sheet given to me, I have been able to ask questions about the study, I have received enough information about the study and I have spoken with:

...............................................................................(name of the resposible researcher)

I understand that my participation is voluntary. I understand that I may withdraw from the study:

1º Any time I want to

2º without having to offer any explanation to

3º without compromising on quality of my healthcare.

I freely agree to participate in this study and I consent to the access and use of my data under the conditions detailed in the information sheet.

- YES
- NOT

**Participant's signature:**

Full name:

**Researcher´s signature**:

**Full name:**

**Date**:

# ANNEXE 3: Health questionare (2 sheets)

**TITLE OF THE STUDY:**

Study of the effect of a nutritional supplement with isoflavones on the symptoms of climacteric in healthy women

**PROMOTER CODE:** PRO_WH_HCT_2016.01

**PROMOTER:** Nektium Pharma SL

**PRINCIPAL INVESTIGATOR:** Dr. Miguel Barber (Baren Clinic), Dr. Ricardo Chirino (ULPGC)

**CENTER:** Baren Clinic

**Person in charge at the center:** Dr. Miguel Barber

**Head of Nektium Pharma SL:** Dra Laura López Ríos

**ANTHROPOMETRIC VARIABLES**

Age: __________

Size: __________

Weight: ___________

Waist circumference: _________________

Hip perimeter: _________________

Blood pressure: _____________________

Resting heart rate: __________________________

Sublingual Temperature: _______________________________

**Questionare (please mark the corresponding option)**

**1.** Let's start by talking about your health. In the last twelve months, would you say that your health has been very good, good, fair, bad or very bad? (Choose an option)

- Very good
- Good
- Regular
- Bad
- Very bad

**2.** Have you been diagnosed with any of the following diseases or health problems?

1. Ginecological cancer: breast, endometrium, cervix.
2. Alergias alimentarias o intolerancias alimenticias: lácteos, huevos, frutos secos, chocolate…
3. Hipertensión arterial
4. Colesterol elevado
5. Diabetes (azúcar elevado)
6. Enfermedades del tiroides
7. Asma, bronquitis crónica o enfisema
8. Enfermedad del corazón
9. Úlcera de estómago
10. Bulimia
11. Anorexia Nerviosa
12. Depression
13. Headaches or migrains
14. Arthrosis or rheumatic problems
15. Osteoporosis

**3.** Indicate if you have any other disease not included in the list above

----------------------------------------------------------------------------------------------------------------------------------------------------------------------------------------------------------------------------------------

**4.-** If questions 2 and / or 3 are affirmative, are you now in treatment for this disease? If yes, what treatment plann?

-----------------------------------------------------------------------------------------------------------------------------

-----------------------------------------------------------------------------------------------------------------------------

---------------------------------------------------------------

**5.** Is there a family history of gynecological cancer (breast, endometrium, cervix)?

□ YES □NO

**6**. When did you have your last period? ……………………………………………………

**7.** How often have you had your last periods? ……………………………….

**8.** Are the last menstrual periods: more abundant than normal or less abundant than normal? ………………………………………

**9.** Are you taking any hormonal treatment (contraceptive pills, hormonal substitute treatment, thyroid treatment, etc.)? If yes, which one?

□YES □NO ………………………………………………………………………………………

**10.** Do you take any other medications regularly? If yes, which one?

□YES □NO …………………………………………………………………………………….

**11**. Are you a smoker?

□YES □Nº Cigarettes/day:………. □ Occasional, only when I go out □NO

**12.-** Do you play any sport? If yes, indicate what type of sport, how often and how long?………………………………………………………………………………………………………………………………………………………………………………………………………………….

***Note:*** The questions 2a, 2b, 2g y 9, if they are positive, they are direct exclusion criteria.

# ANNEXE 4: nutritional guidelines (2 sheets)

**TITLE OF THE STUDY:**

Study of the effect of a nutritional supplement with isoflavones on the symptoms of climacteric in healthy women

**PROMOTER CODE:** PRO_WH_HCT_2016.01

**PROMOTER:** Nektium Pharma SL

**PRINCIPAL INVESTIGATOR:** Dr. Miguel Barber (Baren Clinic), Dr. Ricardo Chirino (ULPGC)

**CENTER:** Baren Clinic

**Person in charge at the center:** Dr. Miguel Barber

**Head of Nektium Pharma SL:** Dra Laura López Ríos

Maintain a balanced diet: including all food groups to get the necessary nutrients.

Eat moderately at each meal and eat 5 meals a day, not letting us get to the main meals very hungry.

Hydration, water: consume a minimum of 1.5l of water a day (6-8 glasses).

Limit consumption: refined flour, white sugar, pastries, sliced bread and industrial juices, as well as soft drinks

Food frequencies / rations by group:

| **Alimento** | **Veces** | **Ración (gr)** | **Ración** |
| --- | --- | --- | --- |
| **Carbohydrates** | 4-6 serv/day |  |  |
| Pasta/rice |  | 60-80 gr | Normal dish |
| Bread |  | 40-60 gr | 3-4 slices or one small bread |
| Potatoes |  | 150-200gr | Big one o 2 small |
| **Greens and vegetables** | More than 2 serv/día | 150-200 gr | Regular portion size |
| **Fruits** | More than 3 serv/día | 120-200 gr | 1 medium slice, 1 cherries cup, strawberry, two melon pieces |
| Pulses | 2-4 serv/weeks | 60-80 gr | Regular portion size |
| **Fish** | 3-4 serv/week | 125-150 gr | 1 steak |
| **Animal proteins** | 3-4 serv/week |  |  |
| Lean meat |  | 100-120 gr | 1 small steak |
| Poultry meat |  | 100-120 gr | ¼ Chicken |
| Eggs |  | 100-120 gr | 1-2 Eggs |
| **Milk and dairy products** | 2-4 serv/day |  |  |
| Milk |  | 200-250 ml | 1 Coup |
| Yoghurt |  | 200-250 gr | 1-2 Yoghurt |
| Cured cheese |  | 40-60 gr | 2-3 Slices |
| Fresh cheese |  | 80-1250 gr | 1 Portion |
| **Nuts** | 3-7 serv/week | 20-30 gr | 1 handful |

**Sausages and fatty meat:** occasional and moderate consumption, Sweets, snacks and soft drinks: occasional and moderate consumption, Butter, margarine or pastries: occasional and moderate consumption, Beer or wine / cider: occasional and moderate consumption.

# ANNEXE 5: Diet questionare (soya intake) (1 sheet)

**TITLE OF THE STUDY:**

Study of the effect of a nutritional supplement with isoflavones on the symptoms of climacteric in healthy women

**PROMOTER CODE:** PRO_WH_HCT_2016.01

**PROMOTER:** Nektium Pharma SL

**PRINCIPAL INVESTIGATOR:** Dr. Miguel Barber (Baren Clinic), Dr. Ricardo Chirino (ULPGC)

**CENTER:** Baren Clinic

**Person in charge at the center:** Dr. Miguel Barber

**Head of Nektium Pharma SL:** Dra Laura López Ríos

1. Do you follow a vegetarian diet?

□YES □NO

1. Do you regularly and consciously consume soy-fortified foods (dairy, soy meat, bean sprouts, etc.)?

□YES □NO

**3.-** Are you allergic to any of the following foods?

Soya o soya derivates: □YES □ NO

Piper (especia): □YES □ NO

Ginger : □YES □ NO

Pomegranate: □YES □ NO

**4.** From the following list of foods indicate how often you eat them several times a day (1). Several times a week (2). Several times a month (3). In an unusual way (4). Never (5)

- Leche de Soja……………………………………………….

- Tofu……………………………………………………….

- Yoghurt/deserts/ice cream/Smoothie of (or containing) soy………….

- Breads/confectionary of (or with) soya flour ……………….

- Soya hamburger…………………………………….

- Soy protein isolate ………………………………

- Soy sauce ………………………………………………

- Raw soybean ……………………………….

- Another food that you think may derive / contain Soy …………………………………

# ANNEXE 6: Cervantes Scale (2 sheets)

**TITLE OF THE STUDY:**

Study of the effect of a nutritional supplement with isoflavones on the symptoms of climacteric in healthy women

**PROMOTER CODE:** PRO_WH_HCT_2016.01

**PROMOTER:** Nektium Pharma SL

**PRINCIPAL INVESTIGATOR:** Dr. Miguel Barber (Baren Clinic), Dr. Ricardo Chirino (ULPGC)

**CENTER:** Baren Clinic

**Person in charge at the center:** Dr. Miguel Barber

**Head of Nektium Pharma SL:** Dra Laura López Ríos

**Cervantes Scale of Health-related quality of life** (Palacios 2004)

Name and surname (initials):________________________________________________

Education level:

🞎 Without studies 🞎 Primary Schooling 🞎 Secundary Education

🞎 Higher education

Birth date: ________________________Current date: __________________________

Please read each of the questions below carefully. You will see that next to 0 and 5 appear some words that represent the two opposite ways of answering the question. In addition, between 0 and 5 there are 4 boxes numbered from 1 to 4. Answer the questions and mark with an X the box that you consider most appropriate according to the degree of agreement between what you think and feel and the proposed answers . In other words, if you totally agree, dial 5 and if you totally disagree, dial 0. If you do not totally agree or disagree, use the intermediate boxes.

Don't think too hard about the answers or spend too much time answering them. Remember that there are no good or bad answers, or cheating answers, and they should all be answered honestly. You may find some questions too personal; Don't worry, remember that this questionnaire is completely anonymous and confidential for the study.

| **Preguntas** | **Valoración** | | | | | | | |
| --- | --- | --- | --- | --- | --- | --- | --- | --- |
| **1. During the day I notice that my head is hurting more and more** | Never | 0 | 1 | 2 | 3 | 4 | 5 | Everyday |
| **2. I'm so nervous that I can't take it anymore** | Never | 0 | 1 | 2 | 3 | 4 | 5 | Constantly |
| **3. I suddenly feel very hot** | Never | 0 | 1 | 2 | 3 | 4 | 5 | In every moment |
| **4. My interest in sex remains as usual** | Much less | 0 | 1 | 2 | 3 | 4 | 5 | Equal or more |
| **5. I can't sleep the necessary hours** | Never happen to me | 0 | 1 | 2 | 3 | 4 | 5 | Constantly |
| **6. Everything bores me, even the things that used to amuse me** | It not true | 0 | 1 | 2 | 3 | 4 | 5 | True |
| **7. I notice tingling in my hands and / or feet** | Not al all | 0 | 1 | 2 | 3 | 4 | 5 | unbearable |
| **8. I consider myself happy in my relationship** | Nothing | 0 | 1 | 2 | 3 | 4 | 5 | Absolutely |
| **9. Suddenly I notice that I start to sweat without having made any effort** | Nevar | 0 | 1 | 2 | 3 | 4 | 5 | Constantly |
| **10. I have lost the ability to relax** | Not al all | 0 | 1 | 2 | 3 | 4 | 5 | Absolutely |
| **11. Although I sleep, I can't rest** | Never happen to me | 0 | 1 | 2 | 3 | 4 | 5 | Constantly |
| **12. I feel like things are spinning around me** | Nothing | 0 | 1 | 2 | 3 | 4 | 5 | considerable |
| **13. My role as a wife or partner is ...** | Nothing significant | 0 | 1 | 2 | 3 | 4 | 5 | Very important |
| **14. I think I retain fluid, because I'm bloated** | No, as always | 0 | 1 | 2 | 3 | 4 | 5 | Yes, much more |
| **15. I am satisfied with my sexual relations** | Not at all | 0 | 1 | 2 | 3 | 4 | 5 | Completely |
| **16. I notice that my muscles or joints hurt** | Not at all | 0 | 1 | 2 | 3 | 4 | 5 | unbearable pain |
| **17. I think others would be better off without me** | Not at all | 0 | 1 | 2 | 3 | 4 | 5 | Sure |
| **18. I'm afraid to make efforts because my urine is leaking** | Not at all | 0 | 1 | 2 | 3 | 4 | 5 | A lot |
| **19. Since I get up I have been tired** | Nothing | 0 | 1 | 2 | 3 | 4 | 5 | A lot |
| **20. I am as healthy as anyone at my age** | Not at all | 0 | 1 | 2 | 3 | 4 | 5 | Equal or better |
| **21. I have a feeling that I'm useless** | Never | 0 | 1 | 2 | 3 | 4 | 5 | All the time |
| **22. I have sex as often as before** | Much less | 0 | 1 | 2 | 3 | 4 | 5 | Igual o más |
| **23. I feel my heart beating very fast and uncontrollably** | Nothing | 0 | 1 | 2 | 3 | 4 | 5 | A lot off |
| **24. Sometimes I think I wouldn't mind being dead** | Never | 0 | 1 | 2 | 3 | 4 | 5 | All the time |
| **25. My health causes me problems with housework** | Not at all | 0 | 1 | 2 | 3 | 4 | 5 | All he time |
| **26. In my relationship I feel treated as equals** | Never | 0 | 1 | 2 | 3 | 4 | 5 | Always |
| **27. I feel itchy in the vagina, as if it were too dry** | Nothing | 0 | 1 | 2 | 3 | 4 | 5 | A lot off |
| **28. I feel depleted** | Never | 0 | 1 | 2 | 3 | 4 | 5 | Always |
| **29.I feel hot flushes** | Never | 0 | 1 | 2 | 3 | 4 | 5 | All the time |
| **30. In my life sex is…** | irrelevant | 0 | 1 | 2 | 3 | 4 | 5 | Extremely important |
| **31. I have noticed that I have more dry skin** | Not, as usual | 0 | 1 | 2 | 3 | 4 | 5 | Yes, much more |

# ANNEXE 7: Follow-up / completion questionnaire (2 Sheets)

**TITLE OF THE STUDY:**

Study of the effect of a nutritional supplement with isoflavones on the symptoms of climacteric in healthy women

**PROMOTER CODE:** PRO_WH_HCT_2016.01

**PROMOTER:** Nektium Pharma SL

**PRINCIPAL INVESTIGATOR:** Dr. Miguel Barber (Baren Clinic), Dr. Ricardo Chirino (ULPGC)

**CENTER:** Baren Clinic

**Person in charge at the center:** Dr. Miguel Barber

**Head of Nektium Pharma SL:** Dra Laura López Ríos

**ANTHROPOMETRIC VARIABLES**

- Weight: _______________________________________________
- Blood pressure: _______________________________________
- waist circumference: __________________________________
- Hip Perimeter: ________________________________________
- Presión arterial: _____________________________________
- Resting heart rate: ___________________________________
- Sublingual temperature: _______________________________

**QUESTIONARE** (thick the option that applies)

**1.** How has your health been in recent weeks in general?

- Very good
- Good
- Regular
- Bad
- Very bad

**2.** Have you had menstruation in the past few weeks? ………………………………………

**3.** Have you had more than one period in the last few weeks? How many? …………………

**4.** If yes, how long have they lasted? …………………………………………….

**5.** Have you taken any alternative treatment to treat climacteric symptoms (birth control pills, hormone replacement therapy, etc.)? If yes, which one?

□YES □NO ………………………………………………………………………………….

**6.-** Have you maintained your eating routine?

□ YES □ NO, I have followed the guideline they have given us □ NO

**7.-** Have you followed your sport routine?

□YES □NO, I have practiced more □ NO, I have practiced less

**8.-** Possible side effects:

Have you been sick in the last few weeks or have you seen a doctor for any condition? If yes, why?

The doctor should indicate if I think there may be a relationship with the treatment. Indicating if the relationship is: Probable, if you consider that there is a relationship between treatment and condition (3), Possible, if you are not clear that the relationship between treatment and condition is direct (2) or if you do not think there is a relationship (1).

**9.-** Concomitant medication. Have you taken any type of medication since you started treatment? If yes: What medication? What for? And how long have you been taking it?

**PHYSICIAN'S ASSESSMENT**

**Based on the answers given by this participant, I consider that she could be receiving:**

A: Placebo 🞎

B: Product 🞎

C. Baren person in charge:

Date

# ANNEXE 8: Phone questionnare (1 sheet)

**TITLE OF THE STUDY:**

Study of the effect of a nutritional supplement with isoflavones on the symptoms of climacteric in healthy women

**PROMOTER CODE:** PRO_WH_HCT_2016.01

**PROMOTER:** Nektium Pharma SL

**PRINCIPAL INVESTIGATOR:** Dr. Miguel Barber (Baren Clinic), Dr. Ricardo Chirino (ULPGC)

**CENTER:** Baren Clinic

**Person in charge at the center:** Dr. Miguel Barber

**Head of Nektium Pharma SL:** Dra Laura López Ríos

**1.** Have you had menstruation in the last week?................................

**2.** Have you slept well in the last week?

YES 🞎 Not 🞎 As usual 🞎

**3**. Have you noticed hot flashes in the last week? If yes, how many? _____________________

4.- How has your health been in recent weeks in general (choose an option)

- Very good
- Good
- Regular
- Bad
- Very bad

**5**.- How has your mood been in the last week?

- Normal:
- Happy:
- Sensitive:
- Irritated:
- Depressed:
- Tired:
- Other: _________________________________________

**6**. Possible side effects:

Have you been sick in the last week or have you seen a doctor for any condition? If yes, why?

*The doctor must indicate if he believes there may be a relationship with the treatment, indicating if the relationship is: Probable, if he considers that there is a relationship between the treatment and the condition (3), Possible, if he does not have of course, the relationship between treatment and illness is direct (2) or if you do not think there is a relationship (1).*

7. Concomitant medication. Have you taken any type of medication? If yes: What medication? What for? And how long have you been taking it?

C. Baren Person in charge:

Date

# ANNEXE 9: TRACKING PERSONAL DIARIES (2 Sheets)

**TITLE OF THE STUDY:**

Study of the effect of a nutritional supplement with isoflavones on the symptoms of climacteric in healthy women

**PROMOTER CODE:** PRO_WH_HCT_2016.01

**PROMOTER:** Nektium Pharma SL

**PRINCIPAL INVESTIGATOR:** Dr. Miguel Barber (Baren Clinic), Dr. Ricardo Chirino (ULPGC)

**CENTER:** Baren Clinic

**Person in charge at the center:** Dr. Miguel Barber

**Head of Nektium Pharma SL:** Dra Laura López Ríos

**COVER:**

- TITLE: Participant diary …………NUMBER: ……….
- SPONSORS: Clínica Baren + Nektium
- DUE DATE: ………………..…
- COLLECTION DATE: ………………………….

**1ª SHEET:**

- INSTRUCCTION OF USE: The diary must be completed daily with the information it requires. It is advisable to carry it with you in your bag or to have it in a place that is easy for you to access, such as the nightstand. The information you must complete is:

- Number of hot flashes per day: Check each time you feel one or complete it at the end of the day

- Mood: Mark the most common mood throughout the day with a number following the attached legend. At most indicate two states of mind.DUDAS:

- *¿What happens if I forget to take the treatment one day?:*
  - Do not worry, take the next dose, the treatment can be prolonged for one more day, but note in observations that day you did not take it.
- *Could the treatment make me feel sick?:*
  - The treatment is a natural product considered as food that should not hurt you. In case you are not feeling well contact your docto
- *The doctor has prescribed me a medication, does it affect the treatment?*
  - Not necessarily, but you should write down what you take and tell the medical staff at the Baren clinic
- MOOD STATE:
- 1. Angry
- 2. Confused
- 3. Sad/deppresed
- 4. Tired
- 5. Sensitive
- 6. Normal
- 7. Happy

**PENULTIMATE SHEET**: Comments and incidences

**LAST SHEET**: Contact details
