# Supplementary material for: Influence of a new botanical combination on quality of life in menopausal Spanish women: Results of a randomized, placebo-controlled pilot study
Source: PLoS One. 2021 Jul 21;16(7):e0255015. doi: 10.1371/journal.pone.0255015 (PMC8294509; doi:10.1371/journal.pone.0255015)
Supplement: S4 File — (DOCX) [file pone.0255015.s004.docx]

PROJECT

Study of the effect of a nutritional supplement containing isoflavones on the symptoms of climacteric in healthy women

**Pro. Code PRO_WH_HCT_2016.01**

INDEX

[I. General information 4](#_Toc473719767)

[1. General purpose: 4](#_Toc473719768)

[2. Development center: 4](#_Toc473719769)

[3. Co-principal investigator from ULPGC: 4](#_Toc473719770)

[4. Co-principal investigator from Clínica Baren: 4](#_Toc473719771)

[5. Sponsor Center 4](#_Toc473719772)

[6. Research team: 4](#_Toc473719773)

[II. Theorical framework 5](#_Toc473719774)

[1. Hormonal patterns 5](#_Toc473719775)

[2. Associated Symptomatology 6](#_Toc473719776)

[3. Standar treatments 6](#_Toc473719777)

[4. Quality of life 7](#_Toc473719778)

[The Greene Climacteric Scale 1982 7](#_Toc473719779)

[Blatt-Kupperman Index (IBK, 1952). 7](#_Toc473719780)

[MENQOL (Menopause quality of life): 7](#_Toc473719781)

[MENCAV (quality scale in menopause). 7](#_Toc473719782)

[Cervantes scale. 7](#_Toc473719783)

[MRS (Menopause Rating Scale) 8](#_Toc473719784)

[5. Phytotherapy 8](#_Toc473719785)

[6. Supplement WH201601 9](#_Toc473719786)

[Glycine max (Soja). 9](#_Toc473719787)

[Punica granatum (Granada). P40P. 10](#_Toc473719788)

[Aframomum melegueta (Granos del paraíso). 10](#_Toc473719789)

[III. Diseño de protocolo. WH_2016 12](#_Toc473719790)

[1. Composition y doses 12](#_Toc473719791)

[2. Objetives 12](#_Toc473719792)

[Primary objetive 12](#_Toc473719793)

[Secundary Objetives 13](#_Toc473719794)

[3. Materials y methods 13](#_Toc473719795)

[Study design 13](#_Toc473719796)

Target [population 13](#_Toc473719797)

[Inclusion Criteria 13](#_Toc473719798)

[Exclusion Criteria 13](#_Toc473719799)

[Statistical Analysis 14](#_Toc473719800)

[Test questionnaires 14](#_Toc473719801)

[Analysis y variables 15](#_Toc473719802)

[4. Protocolo de acción/cronograma 15](#_Toc473719803)

[Visit 1 (V1) 15](#_Toc473719804)

[Visit 2 (V2) 15](#_Toc473719805)

[Visit 3 (V3) 15](#_Toc473719806)

[5. Obtained data 16](#_Toc473719807)

[6. Documentation directory 16](#_Toc473719808)

[7. References 17](#_Toc473719809)

# General information

(COD: PRO_WH_HCT_2016.01)

**INVESTIGATORS IN CHARGE:**

**ULPGC:** RICARDO CHIRINO GODOY, MD PhD

**CLÍNICA BAREN**: MIGUEL A. BARBER, MD PhD

## Study aim:

The aim of the study is to assess the effect of a nutritional supplement based on extracts of plant origin on the quality of life of healthy women over 45 years and with symptoms of climacteric. In addition, a reduction in the number of hot flashes, an improvement in mood and weight loss will be considered.

## Study development centre:

Clínica Baren (Ginematrix). The development centre, Clínica Baren, is a centre specialized in the care of women, in all its phases, from childhood to old age.

## Co-principal investigator from ULPGC:

The co-principal investigator of the project is Ricardo Chirino Godoy, Physician and Professor of Physiology at the ULPGC (Faculty of Health Sciences). Responsible for the project, data analysis and publication of results.

## Co-principal investigator from Clínica Baren:

Miguel A. Barber, gynaecology and obstetrics specialist doctor and president of Ginematrix.

Responsible for the inclusion of participants, medical care and follow-up of the participants during the development of the study.

## Sponsor Centre.

Nektium Pharma SL. (Formerly known as Polinat – Naturals Polyphenols SL).

## Research team:

Laura López Ríos PhD., project manager Nektium Pharma SL. Scientific team: Tanausú Vega PhD., Álvaro Sánchez PhD., Rubén Machín PhD., Julia Wiebe PhD., Miguel Jimenez del Rio PhD.

# Theorical framework

The perimenopausal and menopausal periods constitute an important stage in a woman's life due to the physical and psychosocial changes they generate. The loss of oestrogens and progesterone, key hormones in the fertile life of women, are associated with symptoms that can alter both their physical state and their psychosocial perception.

The Climacteric is known as the transition period that lasts for years, before and after menopause, as a consequence of ovarian exhaustion, associated with a decrease in its ability to produce hormones, follicles and oocytes. The most common age range is usually from 45 to 54 years, with the average age of menopause in our country being 51 years (National Statistics Institute, 2011). It is a period of involution that is accompanied by a series of physical and emotional manifestations related to biological and social changes. All the manifestations are not common to all women who are going through this stage but depend on each woman.

The climacteric includes the phases of perimenopause, menopause and post menopause. Perimenopause is the period that precedes menopause and lasts from the moment the first menstrual cycle changes appear until the year following the definitive cessation of menstruation. It begins with increased vaginal bleeding, followed by a spacing between menses, and last 12 months in a row without menses. The duration can vary between two and five years.

Menopause, the period in a woman's life in which oestrogen is lacking due to the cessation of ovarian activity and, therefore, is no longer fertile, is considered to start after a full year without menstruation (1 year without a period). After a period of one year it considered post-menopause^1^.

The menstrual cycle and hormonal patterns begin to change before the arrival of menopause. During perimenopause, the gradual suppression of ovarian activity occurs, both in quantity and quality of oocytes, making it a period of low fertility.

## Hormonal patterns

The menstrual cycle and hormonal patterns begin to change before the arrival of menopause. During perimenopause, the gradual suppression of ovarian activity occurs, both in quantity and quality of oocytes, making it a period of low fertility.

Plasma decreases in the inhibin B and antimüllerian hormones and an increase in FSH (follicle stimulating hormone) are detected, but these changes do not serve as a diagnosis of perimenopause. Oestradiol levels are kept constant or slightly increased (due to an increase in aromatase activity), while those of progesterone decrease, which can cause spontaneous ovulations, increased bleeding and alteration of menstrual cycles. As perimenopause progresses, oestradiol levels decrease until they are depleted at menopause due to depletion of the ovarian follicles. Thus, serum FSH, oestrogen, and progesterone levels fluctuate (oestrogen and progesterone tend to drop while FSH increases), while LH (luteinizing hormone) levels remain within normal (so they continue synthesizing androgens in the ovary). The increase in FSH levels (≥ 30 IU / L) stimulates ovarian folliculogenesis (especially at the beginning of perimenopause), which occurs at an accelerated rate until menopause. FSH may be indicative of ovarian failure but cannot be used to predict definitive infertility or menopause.

## Associated Symptomatology.

Due to hormonal fluctuation we can find the following symptoms:

**Vasomotor**

• Irregular menstrual periods (increased oestrogen levels may contribute to increased irregular bleeding, swelling, and breast tenderness)

• Disorders in thermoregulation and vasodilation: Hot flashes or hot flashes, night sweats and changes in mood

• Less frequent symptoms: fainting, fatigue or vertigo

**Metabolic**

• Vaginal dryness

• Urinary problems: urinary incontinence and urgency, polyuria and urinary infections, such as cystitis.

• Osteoporosis (especially after menopause, where an accelerated loss of bone mass is observed)

• Muscle and joint discomfort

• Sleep disorders / insomnia

• Mastitis

• Increased risk of contracting cardiovascular diseases not related to age: atherosclerosis and hypertension

• Increased body weight

• Increased risk of getting diabetes

• Increased risk of getting breast and endometrial cancer

**Psychological**

• Irritability, internal restlessness

• Depression, sadness, desire to cry for no apparent reason

• Altered libido / desire, sexual satisfaction

• Anxiety, mental and physical fatigue

## Standard treatments

A large percentage of the female population does not use treatment for vasomotor symptoms, and they alleviate it by following the recommended healthy habits. However, one in four women suffers a significant loss of quality of life, which requires some type of help:

**Hormone replacement therapy**: With oestrogens or combined with progestogens. It is used to combat vasomotor, urogenital, and sexual dysfunction symptoms.

**Selective serotonin reuptake inhibitors** (SSRIs): Recommended for, but not indicated for hot flashes.

**Phytotherapy:** as an alternative to hormone replacement therapy. It is recommended that the preparation have a daily dose of 40-80 phytoestrogens with a minimum of 15 mg genistein.

## Quality of life

According to the WHO, quality of life is defined as “the perception that an individual has of his place in existence, in the context of the culture and the value system in which he lives and in relation to his goals, his expectations, its norms, its concerns”. It is a concept that is influenced by the physical health of the subject, their psychological state, their level of independence, their social relationships, as well as their relationship with their environment. Therefore, to value quality of life of a woman in perimenopause or menopause must take into account both the clinical symptoms (hot flashes, weight gain, insomnia, etc.) and the psychological and social changes they experience (irritability, decreased lividity, etc.).

In order to assess the quality of life of women in the climacteric period, there is a series of questionnaire and scales that collect questions related to the most frequent symptoms:

### Climacteric Greene Scale (The Greene Climacteric Scale) 1982

A 21-item questionnaire that measures several menopausal symptoms on a 4-point Likert scale (0 = “not at all” to 3 = “extremely). Contain three separate sub-scales which measure vasomotor symptoms, somatic symptoms, psychological symptoms, and an additional check out related to sexual function. Psychological symptoms can be further sub-divided to measure anxiety and depression.

### Blatt-Kupperman Menopausal Index (BKI, 1952).

It evaluates 11 symptoms weighted on a scale that ranges from 0 (absence of symptoms) to 3 (severe symptoms); the score obtained allows us to classify the climacteric symptoms into: mild (15-20), moderate (20-35) and severe (> 35)^3^.

### MENQOL (Menopause quality of life):

Proposed by Hilditch et al. in 1996. It identifies 29 symptoms grouped into four domains (vasomotor, psychosocial, physical and sexual). The higher the score, the greater the loss in quality of life.

### MENCAV (Menopause Quality of Life Scale).

It consists of 37 items distributed in 5 dimensions which we call physical health, mental health, family environment, sexual relations and social support. Validated questionnaire, although not applicable. There is a version of the design and validation of the questionnaire in Spanish to measure quality of life in postmenopausal women: the MENCAV questionnaire^4^

### Cervantes Scale.

Original in Spanish and adapted to our population. The scope is Spanish female population between 45 and 64 years old. It consists of 31 items, distributed in the dimensions of menopause and health, which includes vasomotor symptoms, health and aging; sexuality, partner relationship and psychic domain^5^. Its use is requested from the author and granted.

### MRS (Menopause Rating Scale)

It is designed to assess symptoms / complications of age in women under different conditions, to assess the severity of these symptoms and to measure changes before and after menopause replacement therapy. It consists of 11 items that collect the majority of climacteric symptoms/complications and is translated into Spanish^6^.

## Phytotherapy

Phytotherapy consists of the use of medicinal plants and their derivatives for therapeutic purposes. There are many herbal remedies used to mitigate the symptoms of climacteric^7^. Among the most widely used are phytoestrogens, non-steroidal plant chemical compounds that can have actions similar to oestrogens. Among the most commonly used are genistein and daidzein, isoflavones abundant in vegetables such as soybeans (*Glycine max*) or red clover (*Trifolium pratense*).

Overall, the use of isoflavones in menopausal women is associated with an improvement in vasomotor symptoms such as hot flashes, a reduction in the loss of bone mass, an improvement in the lipid profile, improvement in insomnia and mood, although these Effects may vary between ethnic groups.

In the market today there are products designed for the treatment of climacteric. The use of the cimicifuga root and rhizome (*Cimicifuga racemosa* L) in the treatment of premenstrual syndrome, dysmenorrhea, and menopause has been recommended by both the World Health Organization (WHO) and the North American Menopause Society, and the dose tested in the few clinical trials that exist varies between 40-160 mg/day. Red clover (*Trifolium pratense L*) has traditionally been used to treat menopause-related symptoms, although the reduction of flatus or the severity of symptoms at doses between 40-160 mg^7^ has not been clearly demonstrated in clinical trials^7^.

The Spanish Association for the Study of Menopause (AEEM) recommends that the phytoestrogen preparation have a dose between 40-80 mg / day with a minimum of 15 mg of genistein as therapy for alternative use for the treatment of vasomotor symptoms^1^. Adding isoflavones to everyday foods could reduce some of the symptoms. A recent study carried out in Spain, which assesses the impact of soy-fortified beverages (50 mg/day of isoflavones) on the symptoms of climacteric, concluded that the habitual consumption of such beverages improves somatic and urogenital symptoms in those who consumed them. However, this study had, as an important limitation, a low number of participants^8^.

The combination of phytoestrogens with other extracts of plant origin can generate a synergistic effect that improves the symptoms of the climacteric. Su Jin Kang et al, (2015) have recently suggested that the combined effect of pomegranate extract (standardized to ellagic acid) and red clover isoflavones (genistein, biochaine A, formonetin and daizein) improves the symptoms of climacteric and osteoporosis and reduces obesity, suggesting that the antioxidant and inflammatory effect of pomegranate extract potentiates the beneficial effects of red clover isoflavones as oestrogen emulators, although the doses of ellagic acid used in this study are somewhat low^9^.

## Supplement WH201601

Botanical extract based on soy isoflavones (*Glycine max*), ellagic acid from pomegranate (*Punica granatum*) and an 30% alcoholic extract from grains of paradise (*Aframomum melegueta*)

###

### *Glycine max* (Soya).

Main source of isoflavones. Isoflavones are non-steroidal compounds with effects similar to oestrogens, but with less activity. They have been widely used in traditional medicine to treat climacteric symptoms, such as vasomotor problems^7^. Soy has different isoflavone isomers; These include genistein, daidzein, and glycitein as aglycones, and genistin, daizine, and glycitin as glycones. The bioavailability of these molecules depends largely on the intestinal flora, which transforms isoflavones into different active metabolites, for example, daidzein into equol.

The biochemical structure of isoflavones differs from that of steroids, but they share some similarities with oestrogens so that they can bind to oestrogen receptors (ER) and exert similar biological effects, with the affinity of genistein being greater than that of daidzein^10^.

The recommended daily dose is 40 to 80 mg of isoflavones, containing 15 mg of genistein (Spanish Association for the Study of Menopause). The Agency for Healthcare Research and Quality (AHRQ) summarizes, in a meta-analysis on the use of soy isoflavones, that the range of consumed doses of isoflavones per day is 10 -185 mg / dl with an average of 80 mg^11^. In a study carried out with high doses of genistein (60 mg / day for 12 weeks), no side effects were observed, but a significant reduction in the number of hot flashes was observed^12^.

Isoflavone glycones are basically inactive and generally require hydrolysis of their sugar by an intestinal β-glucosidase that converts it to isoflavone aglycone (active compound). A peak of plasma isoflavones is observed 1-2 hours after ingestion, mainly by absorption in the small intestine, and a second peak at 4-8 hours, possibly due to its enterohepatic circulation and absorption in the large intestine. The half-life of isoflavones is 7-9 hours and their absorption rate decline with increasing dose, therefore, to maintain the effect of isoflavones, it is advisable to divide the daily dose into two doses^13^. No serious adverse effects associated with the consumption of isoflavones have been identified at the usual consumption doses^14^.

**Food safety:** Traditionally soy is considered a food and is part of the plants included in the BelFrIt project (project resulting from the efforts of Belgium, France and Italy to harmonize the use of plants in food supplements, guaranteeing safety, the quality and effectiveness of food supplements).

### *Punica granatum* (Pomegranate). P40P.

Pomegranate-rich extract of pomegranate skin and specifically punicalagin. Punicalagins are powerful antioxidants that reduce oxidative stress and prevent lipid peroxidation. Ellagic acid, a type of punicalagin, is associated with a decrease in anti-inflammatory markers and improves the lipid profile. In in vivo studies in ovariectomized rats, an estrogenic effect of pomegranate skin extract has been observed associated with a reduction in osteoporosis^15^. In addition, recent studies have shown the positive effect of punicalagin from pomegranates on improving and strengthening the intestinal microbiota by promoting its growth^16^. Studies in ovariectomized mice with pomegranate extract have concluded that daily intake of pomegranate extract could improve the depressive state and reduce bone loss in menopausal women^17^.

Toxicity studies with extracts of pomegranate (rats) have not revealed any adverse effects at doses of 5 g/Kg by weight (equivalent to 35 g/day in humans), in addition the standardized 30% extract of punicalagins has no toxicity neither acute nor chronic (at 90 days).

*European Regulatory:* Traditionally, pomegranate is a fruit widely consumed throughout the world. Food, including skin, is considered in the BelFrIt List, (project resulting from the efforts of Belgium, France and Italy to harmonize the use of plants in food supplements, guaranteeing the safety, quality and efficacy of food supplements).

### *Aframomum melegueta* (Grains of paradise).

*A. melegueta* is a plant of African origin used as a species and as a remedy to treat stomach pain, diarrhoea and snake bites. The seeds are rich in 6-paradol, 6-gingerol and 6-shogaol, and the aqueous extract of Aframomun melegueta is known to have anti-inflammatory and analgesic properties, in addition to increasing thermogenesis in brown fat in rats^18^ and in humans^19^.

In recent studies conducted by PoliNat in vivo (rats) using electroencephalography (EEG) techniques, we have observed that a 30% alcoholic extract of Aframomun melegueta generates wave patterns similar to those induced by drugs that improve mood and reduce depression. that produce the activation of the serotoninergic and glutamatergic transmissions in the frontal cortex and the hippocampus. Despite there is no scientific bibliography that links Aframomum melegueta with climacteric, we consider that it could be a good candidate to improve the low mood of premenopausal and menopausal participants.

No adverse effects have been described for 30% alcoholic extracts of Aframomun melegueta (extract to be used).

Transient ocular disorders have been observed at a single high dose of 350 mg of grains of paradise (dry amount and much higher than that usually used). Hepatotoxicity that has been observed at high doses of 95% alcoholic extract in rats has also been described as an adverse effect; It is possible that hepatotoxicity is related to the high percentage of alcohol that this extraction has^21^. However, the aqueous extract has been hepatoprotective in rats submitted to a hypercholesterolemic diet^22^. In humans, a 95% alcoholic extract, administered in three daily doses of 40-30-30 mg for four weeks, was not associated with side effects^19,23.^

European Regulatory: Grains of paradise are traditionally used as a condiment in meals and are also considered “Novel food” by the European Commission.

# Protocol design. WH_2016

## Composition and doses.

The formulation is carried out using the concentration of each component recommended in the literature or that used in animal experimentation. With this combination we hope to cover a large number of the symptoms associated with climacteric and we hope that it is enough to improve the quality of life of women in perimenopause and menopause.

From this combination we hope that soy isoflavones improve vasomotor symptoms, as well as hot flashes, *A. melegueta* extract improves mood and punicalagins from pomegranate skin extract improve the bioavailability of isoflavones, enhancing their beneficial effects and helping in improving mood.

| **Compoposition** | **Standardized** | **A.P.** | **Dose/day** | **Dose/Unit intake** |
| --- | --- | --- | --- | --- |
| *Glycine max* | Isoflavones (40%)  Genistein Equivalents, EqvG- (24.73%: Genistin 22.42% y genistein: 1.29% | 40mg Isoflavones 24.73 mg EqvG | 100 mg | 50 mg |
| P40P | Total Punicosides 40 % | 40 mg | 100 mg | 50 mg |
| *Aframomum melegueta* | Hydroalcoholic 30% | 50 mg (extract) | 50 mg | 25 mg |
| Total |  | | 250 mg | 125 mg |

P.A.: Bioactive concentration; EqvG: Genistein equivalents; mg: miligrams;

**Dosage:** Each Capsule contain 125 mg of the product. It will be taken one in the morning and one at night accompanied by food (breakfast and dinner). The treatment will be administered in the form of pills of the same colour and weight between the placebo and the treatment so that they cannot be differentiated on sight**.**

No side effects have been described for any of the components individually or at the recommended doses. It is advisable that the pills be taken with food.

## Objectives

### Main objective

###

To assess whether the combined effect of the three Botanicals extracts improves the quality of life of peri-menopausal and menopausal women.

### Secondary Aims

- To evaluate the improvement in the quality of life for each dimension: menopause and health, psychic, sexuality and partner.
- To assess the potential weight reduction (since Aframomun melegueta has traditionally been used as a fat-burner)

## Materials and Methods

### Study design

Psychometric, double-blind, prospective case-control study. The cases will receive the product and controls the placebo. The treatment will be administered for 8 weeks, and there will be two visits spaced apart: week 1 or start visit and week 8 or end visit (day 56 ± 3). In addition to a phone call one week after the treatment ended (8 ± 3).

The windows of time for the visit and the phone call will be 3 days, before or after the indicated date.

### Target population

Women aged 45-55 years with climacteric phase under medical criteria who come to the Baren Clinic (Las Palmas de GC), sign the informed consent (Annexe 1) and agree to continue treatment for 8 weeks (Annexe 2).

### Inclusion criteria

- Women 45 years of age or older and healthy under medical criteria (The doctor will complete a health questionnaire with them (Annexe 2) and they will be asked for a complete blood and urine analysis.
- Climacteric phase (the symptoms associated with menopause can start from the first menstrual dysfunctions and last up to about 5 years post-menopause): at least 6 months in this phase and not more than 5 years post-menopause.

• Score on the Cervantes Scale (Appendix 6) equal to or greater than 53 points

**Exclusion criteria**

• Having pathologies of hormonal origin

• Surgical menopause (since they lack the classic effects of progressive loss of oestrogens)

• Having a family or own history of cancer of endocrine origin (breast, endometrium, cervix ...)

• Having an allergy / intolerance to dairy or gluten (people with this type of allergies / intolerances tend to substitute allergenic foods for foods rich in soy)

• Have an allergy / intolerance to soy, pomegranate or pepper

• Follow a vegetarian diet or daily and abundant consumption of products rich in soy protein (soy drinks, tofu, soy flour, miso, etc.) during the last year.

• Follow hormone replacement therapy

• Be in treatment with anovulatory

**Statistical analysis**

The quantitative variables will be analysed using the Kolmogorov-Smirnov test to check if they follow a normal distribution. In such case, your data will be presented as the mean ± standard deviation. Otherwise, they will be presented as the median and inter-quartile amplitude. In the case of variables with normal distribution, the differences in the means between the groups or within the group will be analysed by means of the Student's t-test for independent or paired samples, respectively, as well as by ANOVA when more than two groups are included. In the case of quantitative variables that do not follow a normal distribution, the differences of the medians will be analysed using the Mann-Whitney U test for two groups or the Kruskal-Wallis test for more than two groups. Differences in the proportions of the categorical variables will be analysed using the Chi-square test. The correlation of quantitative variables will be analysed by calculating the Pearson or Spearman coefficients for variables that follow or do not follow a normal distribution, respectively.

The sample size will be 36 participants per group (72 participants in total) and is calculated based on a statistical power of 80%, an alpha error of 5%, an improvement in the quality of life scale of the Cervantes scale of 15% and a dropout rate of 10% is contemplated

### Evaluation questionnaires

###

• They will be given a health questionnaire (Annexe 3) and a type of diet (Annexe 4).

• A questionnaire, the Cervantes scale (Annexe 6), will be passed to assess the reduction in the quality of life of women with perimenopause and menopause. It is a questionnaire in Spanish developed specifically for the Spanish population that takes about 10 minutes to complete. The scope of application is the Spanish female population between 45 and 55 years old, consisting of 31 items, distributed in the menopause and health dimensions, which includes vasomotor symptoms, health and aging; sexuality, couple relationship and psychic domain (Annexe 6)

• In the follow-up visit and in the phone call, questions will be included to assess the “adverse effects” and the presence of concomitant medication in case they have become ill during the study (Annexes 6 and 7).

### Analysis and variables

• Two blood tests (general biochemistry and complete blood count) and urine (routine urine including urine sediment study, as well as proteinuria determination) will be performed at the beginning of the study and at the conclusion of the study.

• Both at the beginning of the study and at the conclusion, data will be collected for height, weight, blood pressure, resting heart rate and sublingual temperature,

• They will be given a Cervantes Scale questionnaire at the beginning of the study and at the end of the 8 weeks.

## Protocol od action/schedule

### Visit 1 (V1)

Participants who agree to participate in the study must meet the inclusion criteria (Week 1). In this first visit it will be assessed if they meet the criteria of the Cervantes Scale (scale value above 53 points). If they comply, the procedure will be explained to them and they will sign the informed consent. You will be given 3 cans that will contain a total of 118 pills (the cans will contain 40 capsules each). They will be asked to take two pills a day for 8 weeks. In addition, they will be given a follow-up diary of about 9x9 cm that they must fill out once a day, which they can take with them in their wallet or leave at home. In it they will have to write down the number of hot flashes per day and information regarding their mood. They should start treatment the next day. The physician must complete the V1 questionnaire with each participant. Participants will be encouraged to follow a balanced diet and to continue with the same physical activity that they carry out up to the moment of entering this study.

To verify that the supplement is not causing any discomfort to the participants, a phone call will be made 14-14 days after the start of the supplementation, asking them about their health, if they detect any type of incidents that are not habitual (intolerance, diarrhoea, nausea, muscle pain, etc.) and if they are following supplementation.

### Visit 2 (V2)

###

Follow-up / completion visit (week 8). The cans that were initially delivered with the capsules that were not taken and the follow-up diary will be collected. The doctor will assess how the patient feels, complete the Cervantes Scale questionnaires and the follow-up / completion questionnaires (Health, adverse effects and concomitant medication). The physician must complete the V2 questionnaire with each participant. The participant will be summoned for the last visit (day 56 ± 3).

### Visit 3 (V3)

Phone call (week 9). It will be assessed how the patient feels and if she has noticed anything when leaving the treatment.

## Collected Data

• Initial visit questionnaire (V1): Age, weight, height, BMI, waist circumference and hip circumference, couple situation, physical activity that develops,

• Questionnaire for follow-up visits / study completion (V2)

• Cervantes Scale (V1 and V2) Analytical of each patient. They will be asked to do it before starting the treatment and at the end of it. The results will be delivered to your doctor:

- In blood: complete blood count, globular sedimentation rate, lipid profile, enzymes (ALT, AST, GGT, Alkaline Phosphatase, CK), glucose, urea, creatinine and uric acid.

- In urine: routine urine including sediment analysis, as well as proteinuria determination.

• They will be given a follow-up diary that they must return fill in V2. The objective of the diary is to be able to assess the mood and the number of hot flashes of the participants during the treatment.

## List of documentation.

**Annexe 1**: subject Information sheet (V1)

**Annexe 2:** Informed Consent (V1)

**Annexe 3**: Health questionnaire (V1)

**Annexe 4:** Dietetic counselling (V1)

**Annexe 5:** Dietary questionnaire (to adjust for soy consumption) (V1)

**Annexe 6:** Cervantes Scale (V1 y V2)

**Annexe 7:** Follow-up questionnaire/Completion (V2)

**Annexe 8**: Phone questionnaire (V3)

**Annexe 9**: Follow-up diaries (V1)

| **Questionnaires** | **V1**  **Sem1** | **V2**  **Sem8** | **V3**  **Sem9** | **Doctor** | **Date** | **Participants** | **Date** |
| --- | --- | --- | --- | --- | --- | --- | --- |
| Annexe 1 | X |  |  |  |  |  |  |
| Annexe 2 | X |  |  |  |  |  |  |
| Annexe 3 | X |  |  |  |  |  |  |
| Annexe 4 | X |  |  |  |  |  |  |
| Annexe 5 | X | X |  |  |  |  |  |
| Annexe 6 | X | X |  |  |  |  |  |
| Annexe 7 |  | X |  |  |  |  |  |
| Annexe 8 |  |  | X |  |  |  |  |
| Annexe 9 | X |  |  |  |  |  |  |
| Items | V1  Sem1 | V2  Sem8 | V3  Sem9 | Doctor | Date | Participants | Date |
| analytical request (1) | X |  |  |  |  |  |  |
| analytical request (2) |  | X |  |  |  |  |  |
| Diary delivery | X |  |  |  |  |  |  |
| Bottles 3 delivery | X |  |  |  |  |  |  |
| Diary delivery |  | X |  |  |  |  |  |
| Diary delivery |  | X |  |  |  |  |  |
| Phone call |  |  | X |  |  |  |  |

## References

1. Asociación Española para el Estudio de la Menopausia *AEEM line* (2012).

2. Greene, J. *Maturitas* **29**, 25–31 (1998).

3. C Mascort a, M Beltran i Vilella b, P Solanas a, S Vargas b, S Saura a, C.A. a *Clin. Invest. Ginecol. Obstet.* **35**, (2008).

4. Buendía Bermejoa a, R Rodríguez Segarrab N Yubero Bascuñanab b, V.M.V. c *Atención primaria* **27**, 94–100 (2001).

5. Palacios, S. et al. *Med. Clin. (Barc).* **122**, 205–211 (2004).

6. Heinemann, K. et al. *Health Qual. Life Outcomes* **2**, 45 (2004).

7. Low Dog, T. *Am. J. Med.* **118**, (2005).

8. Tranche, S. et al. *Gynecol. Endocrinol.* **3590**, 1–6 (2016).

9. Kang, S.J. et al. *Nutrients* **7**, 2622–2647 (2015).

10. Commission, F.S., Foods, N. & Committee, E. *Food Saf. Comm. Nov. Foods Expert Comm.* 1–54 (2006).

11. Balk, E. et al. *Evid. Rep. Technol. Assess. (Summ).* **2**, 1–8 (2005).

12. Ferrari, A. *J. Obstet. Gynaecol. Res.* **35**, 1083–1090 (2009).

13. Klein, M., Nahin, R. & Messina, M. *J. Nutr.* 1192S–104S (2010).doi:10.3945/jn.110.121830.exposure

14. Munro, I.C. et al. *Nutr. Rev.* **61**, 1–33 (2003).

15. Satpathy, S., Patra, A. & Purohit, A.P. *Asian Pacific J. Reprod.* **2**, 19–24 (2013).

16. Bialonska D1, Ramnani P, Kasimsetty SG, Muntha KR, Gibson GR, F.D. *Int J Food Microbiol* **140**, 175–82 (2010).

17. Mori-Okamoto, J., Otawara-Hamamoto, Y., Yamato, H. & Yoshimura, H. *J. Ethnopharmacol.* **92**, 93–101 (2004).

18. Ilic, N.M. et al. *J. Agric. Food Chem.* **62**, 10452–10457 (2014).

19. Sugita, J. et al. *Br. J. Nutr.* 1–6 (2013).doi:10.1017/S0007114512005715

20. Igwe, S.A., Emeruwa, I.C. & Modie, J.A. *J. Ethnopharmacol.* **65**, 203–206 (1999).

21. Ilic, N., Schmidt, B.M., Poulev, A. & Raskin, I. *J. Ethnopharmacol.* **127**, 352–356 (2010).

22. Adefegha, S.A., Oboh, G., Adefegha, O.M. & Henle, T. *Pathophysiology* **23**, 191–202 (2016).

23. Sugita, J. et al. *J. Nutr. Sci. Vitaminol. (Tokyo).* **60**, 22–7 (2014).
