# Supplementary material for: Influence of a new botanical combination on quality of life in menopausal Spanish women: Results of a randomized, placebo-controlled pilot study
Source: PLoS One. 2021 Jul 21;16(7):e0255015. doi: 10.1371/journal.pone.0255015 (PMC8294509; doi:10.1371/journal.pone.0255015)
Supplement: S5 File — (DOCX) [file pone.0255015.s005.docx]

PROYECTO

Estudio del efecto de un suplemento nutricional con isoflavonas sobre la sintomatología del climaterio en mujeres sanas

**Cod. Proyecto PRO_WH_HCT_2016.01**

ÍNDICE

[I. Información general 4](#_Toc473719767)

[1. Objetivo del estudio: 4](#_Toc473719768)

[2. Centro de desarrollo: 4](#_Toc473719769)

[3. Coinvestigador principal de la ULPGC: 4](#_Toc473719770)

[4. Coinvestigador principal de la Clínica Baren: 4](#_Toc473719771)

[5. Centro promotor 4](#_Toc473719772)

[6. Equipo de investigación: 4](#_Toc473719773)

[II. Marco teórico 5](#_Toc473719774)

[1. Patrones hormonales 5](#_Toc473719775)

[7. Sintomatología asociada 6](#_Toc473719776)

[8. Tratamientos habituales 6](#_Toc473719777)

[9. Calidad de vida 7](#_Toc473719778)

[Escala climatérica de Greene (The Greene Climacteric Scale) 1982 7](#_Toc473719779)

[Índice de Blatt-Kupperman (IBK, 1952). 7](#_Toc473719780)

[MENQOL (Menopause quality of life): 7](#_Toc473719781)

[MENCAV (Escala de Calidad de Vida en Menopausia). 7](#_Toc473719782)

[Escala Cervantes. 7](#_Toc473719783)

[MRS (Menopause Rating Scale) 8](#_Toc473719784)

[10. Fitoterapia 8](#_Toc473719785)

[11. Suplemento WH201601 9](#_Toc473719786)

[Glycine max (Soja). 9](#_Toc473719787)

[Punica granatum (Granada). P40P. 10](#_Toc473719788)

[Aframomum melegueta (Granos del paraíso). 10](#_Toc473719789)

[III. Diseño de protocolo. WH_2016 12](#_Toc473719790)

[1. Composición y dosis 12](#_Toc473719791)

[2. Objetivo 12](#_Toc473719792)

[Objetivo principal 12](#_Toc473719793)

[Objetivos secundarios 13](#_Toc473719794)

[3. Materiales y métodos 13](#_Toc473719795)

[Diseño del estudio 13](#_Toc473719796)

[Población 13](#_Toc473719797)

[Criterios de inclusión 13](#_Toc473719798)

[Criterios de exclusión 13](#_Toc473719799)

[Análisis estadísticos 14](#_Toc473719800)

[Cuestionarios a valorar 14](#_Toc473719801)

[Análisis y variables 15](#_Toc473719802)

[4. Protocolo de acción/cronograma 15](#_Toc473719803)

[Visita 1 (V1) 15](#_Toc473719804)

[Visita 2 (V2) 15](#_Toc473719805)

[Visita 3 (V3) 15](#_Toc473719806)

[5. Datos que se recogen 16](#_Toc473719807)

[6. Listado de documentación 16](#_Toc473719808)

[7. Referencias 17](#_Toc473719809)

# Información general

(COD: PRO_WH_HCT_2016.01)

**INVESTIGADORES RESPONSABLES**

**ULPGC:** Dr. RICARDO CHIRINO GODOY

**CLÍNICA BAREN**: Dr. MIGUEL A. BARBER

## Objetivo del estudio:

El objetivo del estudio consiste en valorar el efecto de un suplemento nutricional a base de extractos de origen vegetal sobre la calidad de vida de mujeres sanas mayores de 45 años y con sintomatología del climaterio. Además, se valorará una reducción del número de sofocos, una mejora del estado de ánimo y la pérdida de peso.

## Centro de desarrollo:

Clínica Baren (Ginematrix). El centro de desarrollo, Clínica Baren, es un centro especializado en el cuidado de la mujer, en todas sus fases, desde su niñez hasta su vejez.

## Coinvestigador principal de la ULPGC:

El investigador principal del proyecto es el Dr. Ricardo Chirino Godoy, Médico y Profesor de Fisiología de la ULPGC (Facultad de Ciencias de la Salud). Responsable del proyecto, de análisis de datos y de publicación de resultados.

## Coinvestigador principal de la Clínica Baren:

Dr. Miguel A. Barber, médico especialista de ginecología y obstetricia y presidente de Ginematrix.

Responsable de la inclusión de participantes, atención médica y seguimiento de las participantes durante el desarrollo del estudio

## Centro promotor

Nektium Pharma SL. (Antiguamente conocido como Polinat – Polifenoles Naturales SL).

## Equipo de investigación:

Dra. Laura López Ríos, gestora de proyectos Nektium Pharma SL. Equipo científico: Dr. Tanausú Vega, Dr. Álvaro Sánchez, Dr. Rubén Machín, Dr. Julia Wiebe, Dr. Miguel Jimenez del Rio

# Marco teórico

Los periodos perimenopáusico y menopáusico constituyen una etapa importante en la vida de la mujer por los cambios físicos y psicosociales que generan. La pérdida de los estrógenos y de la progesterona, hormonas claves en la vida fértil de La mujer, se asocian con una sintomatología que puede alterar tanto su estado físico como su percepción psicosocial.

El Climaterio se conoce como el periodo de transición que se prolonga durante años, antes y después de la [menopausia](https://es.wikipedia.org/wiki/Menopausia), como consecuencia del agotamiento [ovárico](https://es.wikipedia.org/wiki/Ovario), asociado a una disminución en su capacidad para producir [hormonas](https://es.wikipedia.org/wiki/Hormona), [folículos](https://es.wikipedia.org/wiki/Fol%C3%ADculo_ov%C3%A1rico) y [ovocitos](https://es.wikipedia.org/wiki/Ovocito). La franja de edad más habitual suele ser de los 45 a los 54 años, siendo los 51 años la edad media de la menopausia en nuestro país (Instituto Nacional de Estadística, 2011). Es un período de involución que se acompaña una serie de manifestaciones físicas y emocionales relacionadas con cambios biológicos y sociales. Todas las manifestaciones no son comunes a todas las mujeres que están pasando por esta etapa sino que dependen de cada mujer.

EL climaterio comprende las fases de perimenopausia, menopausia y posmenopausia. La perimenopausia es el periodo que precede a la menopausia y se extiende desde el momento en que aparecen las primeras alteraciones del ciclo menstrual hasta el año siguiente al cese definitivo de la menstruación. Comienza con un aumento del sangrado vaginal, seguido de un espaciamiento entre las menstruaciones y por último 12 meses seguidos sin menstruación. La duración puede variar entre dos y cinco años.

La menopausia, el periodo en la vida de la mujer en el que se carece de estrógenos por el cese de la actividad ovárica y, por tanto ya no es fértil, se considera que se inicia tras un año entero sin menstruación (1 año sin regla). Pasado periodo de un año se habla de posmenopausia1.

El ciclo menstrual y los patrones hormonales empiezan a cambiar antes de la llegada de la menopausia. Durante la perimenopausia se produce la supresión paulatina de la actividad ovárica, tanto en la cantidad como en la calidad de los ovocitos, por lo que es un periodo de baja fertilidad.

## Patrones hormonales

El ciclo menstrual y los patrones hormonales empiezan a cambiar antes de la llegada de la menopausia. Durante la perimenopausia se produce la supresión paulatina de la actividad ovárica, tanto en la cantidad como en la calidad de los ovocitos, por lo que es un periodo de baja fertilidad.

Se detectan descensos plasmáticos de las hormonas inhibina B y antimülleriana y aumento de la FSH (hormona folículo estimulante), pero estos cambios no sirven como diagnóstico de perimenopausia. Los niveles de estradiol se mantienen constantes o ligeramente aumentados (por un incremento en la actividad de la aromatasa), mientras que los de progesterona disminuyen, lo que puede producir ovulaciones espontáneas, aumento del sangrado y alteración de los ciclos menstruales. A medida que avanza la perimenopausia los niveles de estradiol se reducen hasta que se agotan en la menopausia por agotamiento de los folículos ováricos. Por tanto, los niveles séricos de FSH, estrógenos y progesterona fluctúan (estrógenos y progesterona tienden a descender mientras que la FSH aumenta), al tiempo que los niveles de LH (hormona luteinizante) se mantienen dentro de la normalidad (por lo que se siguen sintetizando andrógenos en el ovario).

El aumento en los niveles de FSH (≥ 30 UI/L) estimula la foliculogénesis ovárica (sobre todo al inicio de la perimenopausia), que se produce a ritmo acelerado hasta la menopausia. La FSH puede ser indicativa de insuficiencia ovárica pero no se puede usar para predecir la infertilidad definitiva o la menopausia.

## Sintomatología asociada

Debido a la fluctuación hormonal nos podemos encontrar con los siguientes síntomas:

**Vasomotores**

- Periodos menstruales irregulares (el incremento de los niveles de estrógeno puede contribuir al incremento de sangrados irregulares, hinchazón y sensibilidad mamaria)
- Trastornos en la termorregulación y [vasodilatación](https://es.wikipedia.org/wiki/Vasodilataci%C3%B3n): Bochornos o sofocos, sudoración nocturna y cambios en el estado de ánimo
- Síntomas menos frecuentes: [Desmayo](https://es.wikipedia.org/wiki/S%C3%ADncope), [fatiga](https://es.wikipedia.org/wiki/Cansancio) o [vértigo](https://es.wikipedia.org/wiki/V%C3%A9rtigo)

**Metabólicos**

- Sequedad [vaginal](https://es.wikipedia.org/wiki/Vagina)
- Problemas urinarios: [incontinencia](https://es.wikipedia.org/wiki/Incontinencia_urinaria) y urgencia urinaria, [poliuria](https://es.wikipedia.org/wiki/Poliuria) e [infecciones urinarias](https://es.wikipedia.org/wiki/Infecci%C3%B3n_urinaria), como la [cistitis](https://es.wikipedia.org/wiki/Cistitis).
- [Osteoporosis](https://es.wikipedia.org/wiki/Osteoporosis) (sobre todo tras la menopausia, en donde se observa una pérdida acelerada de masa ósea)
- Molestias musculares y articulares
- [Trastornos](https://es.wikipedia.org/wiki/Insomnio) del sueño/insomnio
- [Mastitis](https://es.wikipedia.org/wiki/Mastitis)
- Mayor riesgo de contraer [enfermedades cardiovasculares](https://es.wikipedia.org/wiki/Cardiopat%C3%ADa) no relacionados con la edad: [aterosclerosis](https://es.wikipedia.org/wiki/Aterosclerosis) e [hipertensión](https://es.wikipedia.org/wiki/Hipertensi%C3%B3n) arterial
- Aumento del peso corporal
- Mayor riesgo de contraer [diabetes](https://es.wikipedia.org/wiki/Diabetes)
- Mayor riesgo de contraer [cáncer de mama](https://es.wikipedia.org/wiki/C%C3%A1ncer_de_mama) y [de endometrio](https://es.wikipedia.org/wiki/C%C3%A1ncer_de_endometrio)

**Psicológicos**

- Irritabilidad, inquietud interna
- Depresión, tristeza, ganas de llorar sin motivo aparente
- Alteración de la libido/deseo, satisfacción sexual
- Ansiedad, cansancio mental y físico

## Tratamientos habituales

Un gran porcentaje de la población femenina no usa tratamiento para la sintomatología vasomotora, y la alivian siguiendo los hábitos saludables recomendados. Sin embargo, una de cada cuatro mujeres ve afectada su calidad de vida por lo que requiere de algún tipo de ayuda:

**Terapia hormonal sustitutoria:** Con estrógenos o combinada con progestágenos. Se emplea para combatir los síntomas vasomotores, los urogenitales y la disfunción sexual.

**Inhibidores selectivos de la recaptación de serotonina (ISRS):** Recomendados para sofocos, aunque no indicado para ellos

**Fitoterapia:** como alternativa a la terapia hormonal sustitutoria. Se recomienda que el preparado tenga una dosis diaria de 40-80 fitoestrógenos con un mínimo de genisteína de 15 mg.

## Calidad de vida

Según la OMS, la calidad de vida se define como “"la percepción que un individuo tiene de su lugar en la existencia, en el contexto de la cultura y del sistema de valores en los que vive y en relación con sus objetivos, sus expectativas, sus normas, sus inquietudes. Se trata de un concepto que está influido por la salud física del sujeto, su estado psicológico, su nivel de independencia, sus relaciones sociales, así como su relación con su entorno.  Por ello, para valorar la calidad de vida de una mujer en perimenopausia o menopausia hay que tener en cuenta tanto la sintomatología clínica (sofocos, ganancia de peso, insomnio, etc.) como los cambios psicológicos y sociales que experimentan (irritabilidad, descenso de la lívido, etc.).

Para poder valorar la calidad de vida las mujeres en el periodo de climaterio, existe una serie de cuestionario y escalas que reúnen preguntas relacionadas con la sintomatología más frecuente:

### Escala climatérica de Greene (The Greene Climacteric Scale) 19982

### Índice de Blatt-Kupperman (IBK, 1952).

Valora 11 síntomas ponderados en una escala que abarca desde el 0 (ausencia de clínica) hasta el 3 (clínica severa); la puntuación obtenida permite clasificar la sintomatología climatérica en: leve (15-20), moderada (20-35) y grave (>35)3.

### MENQOL (Menopause quality of life):

Propuesto por Hilditch y col. en 1996. Identifica 29 síntomas agrupados en cuatro dominios (vasomotor, psicosocial, físico y sexual). Cuanto más alto el puntaje, mayor es la pérdida en la calidad de vida.

### MENCAV (Escala de Calidad de Vida en Menopausia).

Constituida por 37 ítems distribuidos en 5 dimensiones a las que denominamos salud física, salud psíquica, entorno familiar, relaciones sexuales y soporte social. Cuestionario validado, aunque no aplicable. Existe una versión del diseño y la validación del cuestionario en castellano para medir calidad de vida en mujeres posmenopáusicas: el cuestionario MENCAV ^4^

### Escala Cervantes.

Original en castellano y adaptada a nuestra población. El ámbito de aplicación es población española femenina de entre 45 y 64 años. Consta de 31 items, distribuidos en las dimensiones de menopausia y salud, que incluye sintomatología vasomotora, salud y envejecimiento; sexualidad, relación de pareja y dominio psíquico^5^. Solicitado su uso al autor y concedido.

### MRS (Menopause Rating Scale)

Está diseñada para evaluar síntomas/complicaciones de la edad en mujeres bajo diferentes condiciones, para evaluar la severidad de dichos síntomas y para medir cambios pre y post menopausia terapia de reemplazo. Consiste en 11 items que recogen la mayoría de la sintomatología/complicaciones del climaterio y está traducido al castellano^6^.

## Fitoterapia

La fitoterapia consiste en el uso de plantas medicinales y sus derivados con fines terapéuticos. Son muchos los remedios herbales utilizados para mitigar la sintomatología del climaterio^7^. Entre los más utilizados se encuentra los fitoestrógenos, compuestos químicos vegetales no esteroideos que pueden tener acciones similares a los estrógenos. Entre los de uso más habitual se encuentran la genisteína y daidzeína, isoflavonas abundantes en vegetales como la soja (*Glycine max*) o el trébol rojo (*Trifolium pratense*).

De manera general, el uso de isoflavonas en mujeres menopáusicas se asocia con una mejora de la sintomatología vasomotora como son los sofocos, una reducción en la pérdida de masa ósea, una mejora en el perfil lipídico, mejora del insomnio y del ánimo, aunque dichos efectos pueden variar entre etnias.

En el mercado existen hoy en día productos diseñados para el tratamiento del climaterio. El uso de la raíz y el rizoma de cimicífuga (*Cimicifuga racemosa L*) en el tratamiento del síndrome premenstrual, la dismenorrea y la menopausia ha sido recomendado, tanto por la Organización Mundial de la Salud (OMS) como por la Sociedad Norteamericana de la Menopausia, y la dosis probada en los pocos ensayos clínicos que hay varía entre 40-160 mg/día. El trébol rojo (*Trifolium pratense L*) se ha usado tradicionalmente para el tratamiento de los síntomas relacionados con la menopausia aunque no se ha podido demostrar claramente en ensayos clínicos la reducción de los flatos o la severidad de los síntomas a dosis entre 40- 160 mg^7^

La Asociación Española para el Estudio de la Menopausia (AEEM) recomienda que el preparado de fitoestrógenos tenga una dosis entre 40-80 mg/día con un mínimo de genisteína de 15 mg como terapia de uso alternativo para el tratamiento de la sintomatología vasomotora^1^.

La adición de isoflavonas a los alimentos de uso diario podría reducir parte de la sintomatología. Un reciente estudio desarrollado en España, que valora el impacto de las bebidas enriquecidas en soja (50 mg/día de isoflavonas) sobre la sintomatología del climaterio, concluyó que el consumo habitual de dichas bebidas mejora la sintomatología somática y urogenital en quienes las consumían. No obstante, dicho estudio presentaba como limitación importante, un bajo número de participantes^8^.

La combinación de fitoestrógenos con otros extractos de origen vegetal puede generar un efecto sinérgico que mejore la sintomatología del climaterio. Su Jin Kang et al, (2015) han sugerido recientemente que el efecto combinado de extracto de granada (estandarizado a ácido elágico) e isoflavonas de trébol rojo (genisteína, biochaina A, formonetina y daizeina) mejora los síntomas del climaterio y osteoporosis y reduce la obesidad, lo que sugiere que el efecto antioxidante e inflamatorio del extracto de granada potencia los efectos beneficiosos de las isoflavonas del trébol rojo como emuladores de estrógenos, aunque las dosis utilizadas de ácido elágico en este estudio son algo bajas^9^.

## Suplemento WH201601

Extracto natural a base de isoflavonas de soja (*Glycine max*), ácido elágico procedente de la granada (*Punica granatum*) y un extracto alcohólico al 30% de granos del paraiso (*Aframomum melegueta*)

### *Glycine max* (Soja).

Principal fuente de isoflavonas. Las isoflavonas son compuestos no esteroideos con efectos similares a los estrógenos, pero de menor actividad. Han sido ampliamente usados en la medicina tradicional para tratar la sintomatología del climaterio, como los problemas vasomotores ^7^. La soja tiene distintos isómeros de isoflavonas; entre ellos se cuentan la genisteína, la daidzeína y la gliciteína como agliconas y las genistina, daidzina y glicitina en forma de gliconas. La biodisponibilidad de estas moléculas depende en gran medida de la flora intestinal, que transforma las isoflavonas en distintos metabolitos activos, por ejemplo, la daidzeína en equol.

La estructura bioquímica de las isoflavonas difiere de los esteroides pero comparten algunas similitudes con los estrógenos por lo que se pueden unir a receptores estrogénicos (ER) y ejercer efectos biológicos similares, siendo la afinidad de la genisteína mayor que la de la daidzeína^10^.

La dosis diaria recomendada es de 40 a 80 mg de isoflavonas, conteniendo 15 mg de genisteína (Asociación Española para el Estudio de la Menopausia). La Agencia para la calidad e investigación de la asistencia médica (Agency for Healthcare Research and Quality (AHRQ)) resume, en un meta-análisis sobre el uso de isoflavonas de soja, que el rango de dosis consumidas de isoflavonas al día es de 10-185 mg/dl con una media de 80 mg^11^. En un estudio desarrollado con dosis elevadas de genisteína (60 mg/día durante 12 semanas) no se apreciaron efectos secundarios pero sí una reducción significativa del número de sofocos^12^.

Las gliconas isoflavonas son básicamente inactivas y, generalmente requieren la hidrólisis de su azúcar mediante una β-glucosidasa intestinal que la convierte en aglicona de isoflavona (compuesto activo). Se observa un pico de isoflavonas en plasma 1-2 horas tras su ingestión, principalmente por absorción en el intestino delgado, y un segundo pico a las 4-8h, posiblemente por su circulación enterohepática y su absorción en intestino grueso. La vida media de las isoflavonas es de 7-9 horas y su tasa de absorción decrece con el incremento de la dosis, por lo que para mantener el efecto de las isoflavonas se aconseja dividir la dosis diaria en dos tomas^13^. No se han identificado efectos adversos serios asociados al consumo de isoflavonas a las dosis de consumo habitual^14^.

**Seguridad alimentaria**: De manera tradicional la soja se considera un alimento y forma parte de las plantas incluidas en el proyecto BelFrIt (proyecto resultante de los esfuerzos de Bélgica, Francia e Italia para armonizar el uso de las plantas en los complementos alimenticios garantizando la seguridad, la calidad y eficacia de los complementos alimenticios).

### *Punica granatum* (Granada). P40P.

Extracto de piel de granada rico en punicósidos y en concreto en punicalaginas. Las punicalaginas son potentes antioxidantes que reducen el estrés oxidativo y evitan la peroxidación lipídica. El ácido elágico, un tipo de punicalagina, se asocia con un descenso de marcadores anti-inflamatorios y mejora el perfil lipídico. En estudios in vivo en ratas ovariectomizadas, se ha observado un efecto estrogénico del extracto de piel de granada asociado con una reducción de osteoporosis^15^. Además, estudios recientes han puesto de manifiesto el efecto positivo de las punicalaginas procedente de la granada, en la mejora y refuerzo de la microbiota intestinal al favorecer el crecimiento de la misma^16^. Estudios en ratonas ovariectomizadas con extracto de granada han concluido que la ingesta diaria del extracto de granada podría mejorar el estado depresivo y reducir la pérdida ósea en mujeres menopáusicas^17^.

Los estudios de toxicidad con extractos de granada (ratas) no han puesto de manifiesto efectos adversos a dosis de 5 g/Kg por peso (equivalente a 35 g/día en humano), además el extracto estandarizado al 30% de punicalaginas no tiene toxicidad ni aguda ni crónica (a 90 días).

*Regulatorio europeo*: De manera tradicional la granada es una fruta de amplio consumo en el mundo entero. Se considera alimento, incluida la piel, en la BelFrIt List, (proyecto resultante de los esfuerzos de Bélgica, Francia e Italia para armonizar el uso de las plantas en los complementos alimenticios garantizando la seguridad, la calidad y eficacia de los complementos alimenticios).

### *Aframomum melegueta* (Granos del paraíso).

Es una planta de origen africano usada como especie y como remedio para tratar dolor de estómago, diarrea y picaduras de serpiente. Las semillas son ricas en 6-paradol, 6-gingerol y 6-shogaol, y se sabe que el extracto acuoso de *Aframomun melegueta* tiene propiedades antiinflamatorias y analgésicas, además de incrementar la termogénesis en la grasa parda en ratas^18^ y en seres humanos^19^.

En recientes estudios desarrollados por PoliNat in vivo (ratas) utilizando técnicas de electroencefalografía (EEG), hemos observado que un extracto alcohólico de *Aframomun melegueta* al 30% genera patrones de ondas similares a los inducidos por fármacos que mejoran el ánimo y reducen la depresión ya que producen la activación de las transmisiones serotoninérgicas y glutamatérgicas en el córtex frontal y el hipocampo. A pesar de no encontrar bibliografía científica que relacione el *Aframomum melegueta* con el climaterio, consideramos que podría ser un buen candidato para mejorar el estado anímico decaído de las participantes premenopaúsicas y menopaúsicas.

No se han descrito efectos adversos para extractos alcohólicos al 30% de *Aframomun melegueta* (extracto que emplearemos)

A una única dosis elevada de 350 mg de granos del paraíso (cantidad seca y muy superior a la empleada habitualmente) se ha observado trastornos oculares transitorios^20^. También se ha descrito como efecto adverso la hepatotoxicidad que se ha observado a dosis elevadas de extracto alcohólico al 95% en ratas; es posible que la hepatotoxicidad se relacione con el elevado porcentaje de alcohol que tiene dicha extracción^21^. No obstante, el extracto acuoso ha resultado hepatoprotector en ratas sometidas a una dieta hipercolesterolémica^22^. En seres humanos, un extracto alcohólico al 95%, administrado en tres tomas diarias de 40-30-30 mg durante cuatro semanas no se asoció a efectos secundarios^19,23^.

Regulatorio europeo: De manera tradicional los granos del paraíso se usan como condimento en las comidas y además se considera alimento “Novel food” por la Comisión Europea.

# Diseño de protocolo. WH_2016

## Composición y dosis

La formulación se realiza utilizando la concentración de cada componente recomendada en la literatura o la usada en la experimentación animal. Con esta combinación esperamos cubrir un amplio número de la sintomatología asociada al climaterio y esperamos que sea suficiente para mejorar la calidad de vida de las mujeres en perimenopausia y menopausia.

De esta combinación esperamos que las isoflavonas de soja mejoren la sintomatología vasomotora, así como los sofocos, el extracto de aframomum mejore el estado de ánimo y las punicalaginas del extracto de piel de granada mejoren la biodisponibilidad de las isoflavonas potenciando sus efectos beneficiosos y ayude en la mejora del estado de ánimo.

| **Composición** | **Estandarizado** | **P.A** | **Dosis/día** | **Dosis/toma** |
| --- | --- | --- | --- | --- |
| *Glycine max* | Isoflavonas (40%)  Equivalentes de genisteína, EqvG- (24.73%: Genistín 22.42% y genisteína: 1.29% | 40mg Isoflavonas 24.73 mg EqvG | 100 mg | 50 mg |
| P40P | Punicosidos totales 40 % | 40 mg | 100 mg | 50 mg |
| *Aframomum melegueta* | Hidroalcohólico 30% | 50 mg (extracto) | 50 mg | 25 mg |
| Total |  | | 250 mg | 125 mg |

P.A.: concentración de principio activo; EqvG: equivalentes de genisteína; mg: miligramos;

**Dosificación**: Cápsulas que contendrán 125 mg del producto. Se tomarán una por la mañana y otra por la noche acompañadas de comida (desayuno y cena). El tratamiento se administrará en forma de píldoras de igual color y peso entre el placebo y el tratamiento de manera que no puedan ser diferenciadas a la vista.

No se han descrito efectos secundarios para ninguno de los componentes de manera individual ni a las dosis recomendadas. Es aconsejable que las pastillas se tomen acompañadas de comida.

## Objetivo

### Objetivo principal

###

Evaluar si el efecto combinado de los tres extractos vegetales mejora la calidad de vida de mujeres peri-menopaúsicas y menopaúsicas.

### Objetivos secundarios

- Evaluar la mejora en la calidad de vida por cada dimensión: menopausia y salud, psíquica, sexualidad y pareja.
- Evaluaremos la reducción de peso (ya que el *Aframomun melegueta* ha sido usado tradicionalmente como quema-grasas)

## Materiales y métodos

### Diseño del estudio

Estudio psicométrico, prospectivo de casos y controles, doble ciego aleatorizado. Los casos recibirán el producto y los controles placebo. El tratamiento se administrará durante 8 semanas, y se realizarán dos visitas espaciadas: semana 1 o visita de inicio y semana 8 o visita de finalización (día 56 ± 3). Además de una llamada telefónica una semana después de haber concluido el tratamiento (8 ± 3).

Las ventanas temporales para la visita y la llamada telefónica serán de 3 días, antes o después de la fecha señalada.

### Población

Mujeres de 45-55 años con fase de climaterio bajo criterio médico que acudan a la Clínica Baren (Las Palmas de GC), que firmen el consentimiento informado (Anexo 1) y estén de acuerdo en seguir el tratamiento durante 8 semanas (Anexo 2).

### Criterios de inclusión

- Mujeres de 45 años o mayores y sanas bajo criterio médico (El médico completará con ellas un cuestionario de salud (anexo 2) y se les solicitará un análisis de sangre y orina completos).
- Fase de climaterio (la sintomatología asociada a la menopausia puede empezar desde las primeras disfunciones de la menstruación y durar hasta unos 5 años posmenopausia): al menos 6 meses en dicha fase y no más de 5 años de postmenopausia.
- Con una puntuación en la Escala Cervantes (anexo 6) igual o mayor a 53 puntos

### Criterios de exclusión

- Tener patologías de origen hormonal
- Menopausia quirúrgica (dado que carecen de los efectos clásicos por pérdida progresiva de estrógenos)
- Tener antecedentes familiares o propios de cáncer de origen endocrino (mama, endometrio, cérvix...)
- Tener alergia/intolerancia a los lácteos o al gluten (las personas con este tipo de alergias/intolerancias tienden a sustituir los alimentos alergénicos por alimentos ricos en soja)
- Tener alergia/intolerancia a la soja, la granada o a la pimienta
- Seguir una dieta vegetariana o consumo diario y abundante de productos ricos en proteínas de soja (bebidas de soja, tofu, harina de soja, miso, etc.) durante el último año.
- Seguir tratamiento hormonal sustitutorio
- Estar en tratamiento con anovulatorios

### Análisis estadísticos

Las variables cuantitativas serán analizadas mediante el test de Kolmogorov-Smirnov para comprobar si siguen una distribución normal. En tal caso, sus datos se presentarán como la media ± desviación estándar. En caso contrario, se presentarán como la mediana y la amplitud inter-cuartil. En el caso de variables con distribución normal, las diferencias de las medias entre los grupos o intra-grupo se analizarán mediante la prueba t de Student para muestras independientes o pareadas, respectivamente, así como mediante ANOVA cuando se incluyan más de dos grupos. En el caso de variables cuantitativas que no sigan una distribución normal, las diferencias de las medianas se analizarán mediante el test U de Mann-Whitney para dos grupos o el de Kruskal-Wallis para más de dos grupos. Las diferencias en las proporciones de las variables categóricas se analizarán mediante el test de Chi-cuadrado. La correlación de variables cuantitativas se analizará mediante el cálculo de los coeficientes de Pearson o de Spearman para variables que sigan o no sigan una distribución normal, respectivamente.

El tamaño muestral será de 36 participantes por grupo (72 participantes en total) y se calcula en base a un poder estadístico del 80%, un error alfa del 5%, una mejoría en la baremación de la calidad de vida de la escala Cervantes de un 15% y se contempla un porcentaje de abandono del 10%

### Cuestionarios a valorar

###

- Se les pasará un cuestionario de salud (Anexo 3) y de tipo de alimentación (Anexo 4).
- Se pasará un cuestionario, la escala Cervantes (Anexo 6), que valorara la reducción de la calidad de vida de mujeres con perimenopausia y menopausia. Es un cuestionario en castellano desarrollado específicamente para población española que llevar unos 10 minutos realizarlo. El ámbito de aplicación es población española femenina de entre 45 y 55 años, consta de 31 items, distribuidos en las dimensiones menopausia y salud, que incluye sintomatología vasomotora, salud y envejecimiento; sexualidad, relación de pareja y dominio psíquico (Anexo 6)
- En la visita de seguimiento y en la llamada telefónica se incluirá preguntas para valorar los “efectos adversos” y la presencia de medicación concomitante en caso de que se hayan puesto malas en el transcurso del estudio (Anexos 6 y 7).

### Análisis y variables

- Se les realizarán dos análisis de sangre (bioquímica general y hemograma completo) y de orina (rutinario de orina incluyendo estudio del sedimento de orina, así como determinación de proteinuria) al inicio del estudio y a la conclusión del mismo.
- Tanto al inicio del estudio como a la conclusión de recogerán datos de altura, peso, presión arterial, frecuencia cardiaca en reposo y temperatura sublingual,
- Se les pasará cuestionario de la Escala Cervantes al inicio del estudio y al final de las 8 semanas.

## Protocolo de acción/cronograma

### Visita 1 (V1)

Las participantes que accedan participar en el estudio deberán cumplir los criterios de inclusión (Semana 1). En esta primera visita se valorará sí cumplen con el criterio de la Escala Cervantes (valor de escala por encima de 53 puntos). En caso de que lo cumplan se les explicará el procedimiento y firmarán el consentimiento informado. Se les entregará 3 botes que contendrán en total 118 pastillas (los botes contendrán 40 cápsulas cada uno). Se les pedirá que se tomen dos pastillas al día durante 8 semanas. Además, se les entregará un diario de seguimiento de unos 9x9 cm que deberán rellenar una vez al día, el cual podrán llevar consigo en la cartera o dejar en sus casas. En él tendrán que anotar el número de sofocos por día e información concerniente a su estado de ánimo. Deberán empezar con el tratamiento al día siguiente. El médico deberá completar el cuestionario de V1 con cada participante. A las participantes se les animará a seguir una alimentación equilibrada y a que continúen con la misma actividad física que realizan hasta el momento de entrar en este estudio.

Para comprobar que el suplemento no está causando ninguna molestia a las participantes, a los 14-16 días de inicio de la suplementación se les hará una **llamada telefónica** en la que se les preguntará por su estado de salud, si detecta cualquier tipo de incidencias no habituales (intolerancia, diarreas, náuseas, dolores musculares, etc) y si están siguiendo la suplementación.

### Visita 2 (V2)

###

Visita de seguimiento/finalización (semana 8). Se recogerán los botes que se les entregó inicialmente con las cápsulas que no se hayan tomado y el diario de seguimiento. El médico valorará cómo se siente la paciente, completará los cuestionarios de Escala Cervantes y los cuestionarios de seguimiento/finalización (Salud, efectos adversos y medicación concomitante). El médico deberá completar el cuestionario de V2 con cada participante. Se citará a la participante para la última visita (día 56±3).

### Visita 3 (V3)

Llamada telefónica (semana 9). Se valorará cómo se siente la paciente y si ha notado algo al dejar el tratamiento.

## Datos que se recogen

- Cuestionario de visitas de inicio (V1): Edad, peso, talla, IMC, perímetro de cintura y perímetro de cadera, situación de pareja, actividad física que desarrolla,
- Cuestionario de visitas de seguimiento/finalización de estudio (V2)
- Escala Cervantes (V1 y V2)
- Analítica de cada paciente. Se les pedirá la realicen antes de empezar con el tratamiento y al finalizarlo. Los resultados se entregarán a su médico:
  - En sangre: hemograma completo, velocidad de sedimentación globular, perfil lipídico, enzimas (ALT, AST, GGT, Fosfatasa alcalina, CK), glucosa, urea, creatinina y ácido úrico.
  - En orina: rutinario de orina incluyendo análisis de sedimento, así como determinación de proteinuria.
- Se les entregará un diario de seguimiento que deberán devolver relleno en la V2. El objetivo del diario es poder evaluar el estado anímico y el número de sofocos de las participantes mientras dure el tratamiento.

## Listado de documentación

**Anexo 1**: Hoja de información al paciente (V1)

**Anexo 2:** Consentimiento informado (V1)

**Anexo 3**: Cuestionario de salud (V1)

**Anexo 4:** Recomendaciones dietéticas (V1)

**Anexo 5:** Cuestionario de alimentación (Para ajustar por consumo de soja) (V1)

**Anexo 6:** Escala Cervantes (V1 y V2)

**Anexo 7:** Cuestionario seguimiento/finalización (V2)

**Anexo 8**: Cuestionario telefónico (V3)

**Anexo 9**: Diarios de seguimiento (V1)

| **Cuestionarios** | **V1**  **Sem1** | **V2**  **Sem8** | **V3**  **Sem9** | **Médico** | **Fecha** | **Participante** | **Fecha** |
| --- | --- | --- | --- | --- | --- | --- | --- |
| Anexo 1 | X |  |  |  |  |  |  |
| Anexo 2 | X |  |  |  |  |  |  |
| Anexo 3 | X |  |  |  |  |  |  |
| Anexo 4 | X |  |  |  |  |  |  |
| Anexo 5 | X | X |  |  |  |  |  |
| Anexo 6 | X | X |  |  |  |  |  |
| Anexo 7 |  | X |  |  |  |  |  |
| Anexo 8 |  |  | X |  |  |  |  |
| Anexo 9 | X |  |  |  |  |  |  |
| Items | V1  Sem1 | V2  Sem8 | V3  Sem9 | Médico | Fecha | Participante | Fecha |
| Solicitud analítica (1) | X |  |  |  |  |  |  |
| Solicitud analítica (2) |  | X |  |  |  |  |  |
| Diario entrega | X |  |  |  |  |  |  |
| Botes 3 entrega | X |  |  |  |  |  |  |
| Diario recogida |  | X |  |  |  |  |  |
| Botes recogida |  | X |  |  |  |  |  |
| Llamada telefónica |  |  | X |  |  |  |  |

## Referencias

1. Asociación Española para el Estudio de la Menopausia *AEEM line* (2012).

2. Greene, J. *Maturitas* **29**, 25–31 (1998).

3. C Mascort a, M Beltran i Vilella b, P Solanas a, S Vargas b, S Saura a, C.A. a *Clin. Invest. Ginecol. Obstet.* **35**, (2008).

4. Buendía Bermejoa a, R Rodríguez Segarrab N Yubero Bascuñanab b, V.M.V. c *Atención primaria* **27**, 94–100 (2001).

5. Palacios, S. et al. *Med. Clin. (Barc).* **122**, 205–211 (2004).

6. Heinemann, K. et al. *Health Qual. Life Outcomes* **2**, 45 (2004).

7. Low Dog, T. *Am. J. Med.* **118**, (2005).

8. Tranche, S. et al. *Gynecol. Endocrinol.* **3590**, 1–6 (2016).

9. Kang, S.J. et al. *Nutrients* **7**, 2622–2647 (2015).

10. Commission, F.S., Foods, N. & Committee, E. *Food Saf. Comm. Nov. Foods Expert Comm.* 1–54 (2006).

11. Balk, E. et al. *Evid. Rep. Technol. Assess. (Summ).* **2**, 1–8 (2005).

12. Ferrari, A. *J. Obstet. Gynaecol. Res.* **35**, 1083–1090 (2009).

13. Klein, M., Nahin, R. & Messina, M. *J. Nutr.* 1192S–104S (2010).doi:10.3945/jn.110.121830.exposure

14. Munro, I.C. et al. *Nutr. Rev.* **61**, 1–33 (2003).

15. Satpathy, S., Patra, A. & Purohit, A.P. *Asian Pacific J. Reprod.* **2**, 19–24 (2013).

16. Bialonska D1, Ramnani P, Kasimsetty SG, Muntha KR, Gibson GR, F.D. *Int J Food Microbiol* **140**, 175–82 (2010).

17. Mori-Okamoto, J., Otawara-Hamamoto, Y., Yamato, H. & Yoshimura, H. *J. Ethnopharmacol.* **92**, 93–101 (2004).

18. Ilic, N.M. et al. *J. Agric. Food Chem.* **62**, 10452–10457 (2014).

19. Sugita, J. et al. *Br. J. Nutr.* 1–6 (2013).doi:10.1017/S0007114512005715

20. Igwe, S.A., Emeruwa, I.C. & Modie, J.A. *J. Ethnopharmacol.* **65**, 203–206 (1999).

21. Ilic, N., Schmidt, B.M., Poulev, A. & Raskin, I. *J. Ethnopharmacol.* **127**, 352–356 (2010).

22. Adefegha, S.A., Oboh, G., Adefegha, O.M. & Henle, T. *Pathophysiology* **23**, 191–202 (2016).

23. Sugita, J. et al. *J. Nutr. Sci. Vitaminol. (Tokyo).* **60**, 22–7 (2014).
